# Scalable modeling of multi-spin ensembles in SABRE hyperpolarization: a symmetry-based framework for zero and ultralow fields

**DOI:** 10.5194/mr-7-53-2026

**Published:** 2026-05-21

**Authors:** Danil Markelov, Alexander Snadin, Alexey Kiryutin, Danila Barskiy, Alexandra Yurkovskaya

**Affiliations:** 1 International Tomography Center SB RAS, Institutskaya 3a, Novosibirsk, 630090, Russia; 2 Frost Institute for Chemistry and Molecular Science, Department of Chemistry, University of Miami, Coral Gables, FL 33146, USA

## Abstract

This work presents a theoretical framework for quantitative, scalable modeling of signal amplification by reversible exchange (SABRE) experiments under zero- and ultralow-field (ZULF) conditions. SABRE exploits the singlet spin order of parahydrogen to hyperpolarize nuclear spins of substrates without chemical modification, enhancing NMR signals. In the ZULF SABRE method, polarization transfer occurs in ultralow magnetic fields where Zeeman interactions are comparable to or weaker than scalar couplings, enabling coherent mixing of spin states and revealing interactions often suppressed at high fields. Our approach captures the full quantum dynamics of SABRE, including coherent evolution, chemical exchange, and relaxation, within a Liouville space formalism. We demonstrate that the Hamiltonian, relaxation, and exchange superoperators possess symmetry with respect to the total spin, allowing the dynamics to be rigorously restricted to the zero-quantum coherence subspace. This symmetry-based reduction yields a scalable framework for efficient simulation of multi-spin SABRE systems, allowing the treatment of arbitrary spin ensembles, including those containing ${}^{15}N$ , ${}^{13}C$ , ${}^{1}H$ , and other nuclei. The approach is validated against full Liouville space calculations for small systems and is further applied to a 14-spin SABRE complex, demonstrating its ability to treat spin systems of a complexity well beyond the reach of conventional full Liouville space simulations. The framework thus provides a predictive tool for optimal polarization fields, ZULF NMR spectra, and the design of novel hyperpolarization experiments.

## 1 Introduction

Nuclear magnetic resonance (NMR) is a powerful spectroscopic technique that provides detailed information about molecular structure and dynamics. Its sensitivity, however, is fundamentally limited by the low thermal polarization of nuclear spins, a limitation that is particularly severe for heteronuclei whose low gyromagnetic ratios and natural abundances result in intrinsically weak NMR signals (Levitt, 2008). Enhancing the polarization levels, i.e. hyperpolarization, of the magnetic nuclei is therefore of central importance for extending the applicability of NMR to chemically and biologically relevant systems (Eills et al., 2023; Petersen et al., 2025; McBride et al., 2025; Kuhn et al., 2023; Zachrdla et al., 2025; Cavallari et al., 2018; Angelovski et al., 2023; Keshari and Wilson, 2014; Boutin et al., 2011).

**Figure 1:**
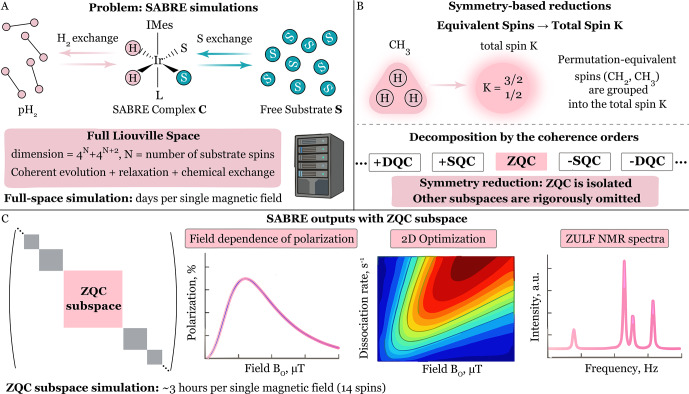
Conceptual overview of the symmetry-based framework for SABRE under zero- and ultralow-field (ZULF) NMR conditions. **(A)** The full Liouville space has a dimension of $4^{N}$ , where N is the number of spins, rendering simulations of SABRE that include coherent spin evolution, relaxation, and chemical exchange computationally demanding. **(B)** Two symmetry reductions are employed: (1) permutation symmetry of equivalent spins, which is exploited via the introduction of effective spins K , and (2) conservation of the total spin projection $F_{z}$ , which allows the dynamics to be rigorously restricted to the zero-quantum coherence (ZQC) subspace. **(C)** As a result, the problem is reduced to a substantially smaller ZQC Liouville space representation that still fully captures the relevant spin dynamics, thereby enabling quantitative simulations of multi-spin SABRE systems.

Among the available hyperpolarization techniques, signal amplification by reversible exchange (SABRE) has emerged as a versatile and experimentally accessible method for boosting NMR sensitivity (Adams et al., 2009; Rayner and Duckett, 2018; Buntkowsky et al., 2022; Pravdivtsev et al., 2021; Atkinson et al., 2009). In SABRE, the spin order of parahydrogen is transferred to a target substrate through reversible binding to a transient metal–ligand complex, without permanent chemical modification of the molecule, as shown in Fig. 1A (Barskiy et al., 2019; Pravdivtsev et al., 2013; Shchepin et al., 2019). Importantly, SABRE has been shown to efficiently hyperpolarize a variety of heteronuclear spin systems, including ${}^{13}C$ -, ${}^{15}N$ -, ${}^{19}F$ -, ${}^{31}P$ -, and ${}^{77}\mathrm{Se}$ -containing compounds (Kozinenko et al., 2025; Vaneeckhaute et al., 2023; Svyatova et al., 2019; Schmidt et al., 2023; Kiryutin et al., 2025; Markelov et al., 2024; Kiryutin et al., 2018; Burns et al., 2015; Olaru et al., 2018; Markelov et al., 2025; TomHon et al., 2025; Zheng et al., 2026). In biological NMR, heteronuclear detection is especially advantageous, as proton-based experiments are limited by the strong water background and short relaxation times (Mishra et al., 2016; Cho et al., 2019; Kim et al., 2025; Dey et al., 2020; Park and Wang, 2022).

Importantly, the efficiency of SABRE hyperpolarization is governed by the coherent spin dynamics within the coupled spin network formed by the parahydrogen-derived hydrides and the nuclear spins of the substrate in the transient metal complex (Pravdivtsev et al., 2013; Pravdivtsev et al., 2015; Ivanov et al., 2014; Pravdivtsev et al., 2014; Pravdivtsev et al., 2014; Pravdivtsev et al., 2014; Kiryutin et al., 2013). Despite growing experimental interest in SABRE, the number of rigorous theoretical models describing its spin dynamics remains relatively limited. In the present work, we adopt a fully rigorous description of SABRE spin dynamics based on a Liouville space formulation, in which coherent evolution, relaxation, and chemical exchange are treated on equal footing through a master equation. Although alternative approaches exist (Lindale et al., 2020), our focus here is specifically on a framework that captures the complete quantum dynamics of the system (Knecht et al., 2020; Knecht and Ivanov, 2019; Knecht et al., 2016). This approach is firmly rooted in the standard theory of NMR spin dynamics (Limbach, 1991; Abergel and Palmer, 2005; Cuperlovic et al., 2000; Zaiss and Bachert, 2013; Schwartz et al., 1982; Jameson and Bruschweiler, 2021) and provides a conceptually transparent description of SABRE polarization transfer. However, the dimensionality of Liouville space grows exponentially with the number of spins involved, making direct simulations computationally intractable for large heteronuclear SABRE systems; see Fig. 1A. Consequently, the development of a general and scalable framework for such multi-spin ensembles remains a significant challenge. Motivated by this, we present in this work a complementary approach to recent efforts (Mamone et al., 2025) that leverages symmetry to render the full Liouville space treatment computationally tractable for complex SABRE systems.

In parallel with the development of hyperpolarization methods, zero- and ultralow-field (ZULF) NMR has been gaining increasing attention as a rapidly developing complement to conventional high-field NMR that enables access to spin dynamics and interactions inaccessible at high magnetic fields (Blanchard and Budker, 2016; Blanchard et al., 2020; Ledbetter et al., 2011; Ledbetter et al., 2009; Put et al., 2021; Kiryutin et al., 2021; Barskiy et al., 2025; Barskiy et al., 2019; Xu et al., 2026; Sheberstov et al., 2021). In this regime, experiments are performed in magnetic fields where Zeeman interactions are comparable to or weaker than scalar J couplings. ZULF NMR therefore offers several distinctive advantages that have driven its increasing adoption. The absence of a strong static magnetic field eliminates line broadening due to magnetic field inhomogeneities and enables exceptionally high spectral resolution (Blanchard et al., 2013; Picazo-Frutos et al., 2024; Sjolander et al., 2020). Moreover, ZULF NMR is inherently compatible with compact, magnetically shielded setups and non-inductive detection schemes based on optically pumped magnetometers, allowing high sensitivity without the need for superconducting magnets (Ledbetter et al., 2009; Jiang et al., 2019; Hong et al., 2025). For heteronuclear systems in particular, ZULF NMR provides access to spin interactions and dynamical effects that are strongly suppressed or effectively truncated at high magnetic fields (Blanchard et al., 2015; Teleanu et al., 2025; Pravdivtsev et al., 2013). In the ZULF regime, heteronuclear spin evolution is governed by the full, non-secular spin Hamiltonian, allowing otherwise negligible coupling terms and coherent mixing of spin states to influence the observed spectra (Kiryutin et al., 2021; Zhukov et al., 2020; Zhukov et al., 2021). This makes ZULF NMR especially well-suited for probing heteronuclear spin dynamics beyond the high-field approximation.

The combination of the parahydrogen-based hyperpolarization with zero- and ultralow-field NMR provides a powerful approach for enhancing the sensitivity of NMR detection (Theis et al., 2012; Theis et al., 2011; Buckenmaier et al., 2025; Rodriguez et al., 2025; Van Dyke et al., 2022; Put et al., 2023). By leveraging hyperpolarization in the ultralow-field regime, enhanced signals can be observed from collective spin states comprising ${}^{1}H$ , ${}^{13}C$ , ${}^{15}N$ , and other nuclei. The theoretical consideration of SABRE under the ZULF conditions, or ZULF SABRE, is helpful for guiding the design of experiments, estimating optimal fields for polarization buildup, and predicting ZULF NMR spectra, thereby supporting the development of new polarization transfer strategies and their adaptation to the unique conditions of SABRE systems.

Theoretical and computational studies of SABRE spin dynamics have attracted significant attention in recent years. In a number of works, it has been shown that the key contributions to polarization transfer in SABRE can be understood in terms of zero-quantum operators, highlighting the central role of the zero-quantum coherence (ZQC) subspace (Eriksson et al., 2022; Snadin et al., 2024; Markelov et al., 2021; Li et al., 2022). In Liouville space, this subspace is defined by operators with zero eigenvalue of the superoperator of the z projection of the total spin, ${\hat{\hat{F}}}_{z}$ , where the quantization axis is set by the direction of the ultralow external magnetic field. In particular, Mamone et al. (2025) demonstrated that the description in terms of zero-quantum operators, including exchange and relaxation, successfully captures SABRE dynamics while reducing the dimensionality of the problem.

Here, we place these observations on a rigorous footing. We formally show (Fig. 1B) that the Hamiltonian, relaxation, and chemical exchange superoperators in Liouville space possess a well-defined symmetry with respect to ${\hat{\hat{F}}}_{z}$ . This symmetry guarantees that the dynamics is restricted to the ZQC subspace and that only these components contribute to polarization transfer in ZULF SABRE. Exploiting this symmetry, we develop a general and scalable computational framework based on systematic restriction to the ZQC subspace (Fig. 1C). The approach is validated against full Liouville space simulations for small spin systems, reproducing exact results while providing computational speedups of 30–50 times. For larger systems, such as a 14-spin SABRE complex, the method enables simulations that would be computationally infeasible in the full Liouville space.

## 2 Theory

### 2.1 Block-diagonal decomposition of Hilbert space

We begin by considering the low-field spin Hamiltonian governing the coherent dynamics of a coupled N -spin system (in units of ℏ ):

1 $$\hat{H}(t)=-B_{0}(t)\sum_{l=1}^{N}\gamma^{l}{\hat{I}}_{z}^{\;l}+2\pi \sum_{l<m}^{N}J^{lm}({\hat{I}}^{\;l}\cdot {\hat{I}}^{\;m}),$$

where N is the total number of nuclei in the spin system, $B_{0}(t)$ is an external magnetic field, $\gamma^{l}$ is the gyromagnetic ratio of nucleus l , $J^{lm}$ is the J -coupling constant between nuclei l and m , and $\left({\hat{I}}^{\;l}\cdot {\hat{I}}^{\;m}\right)$ denotes the scalar product of the corresponding spin operators. At ultralow magnetic fields, chemical shift terms are neglected. However, the framework presented below is not limited to this regime; chemical shifts can be incorporated straightforwardly without modifying the theoretical description.

In this section, we analyze the symmetry properties of the Hamiltonian in Eq. (1) and show that they give rise to a block-diagonal structure in an appropriate basis. As a result, the spin dynamics can be rigorously restricted to invariant subspaces of reduced dimensionality. This symmetry-based reduction provides a practical route for treating large, coupled spin systems and underpins the SABRE applications presented in the subsequent sections.

Importantly, throughout this work we treat the general and experimentally relevant case of a non-zero magnetic field, $B_{0}\neq 0$ . In practice, a strictly zero field is never realized due to residual magnetic fields, which makes the present approach directly applicable to SABRE polarization transfer at ultralow fields and to the simulation of ZULF NMR spectra across different coupling regimes, ranging from the J -coupling-dominated to the Zeeman-dominated limit (Buckenmaier et al., 2025). A separate and careful symmetry analysis would be required only in the idealized limit $B_{0}=0$ , which lies beyond the scope of the present study.

#### 2.1.1 The group of magnetically equivalent nuclei

Assume that we have a group $G_{P}$ of P magnetically equivalent spins, reflecting the underlying molecular symmetry, for instance, the three protons of a methyl ( ${\mathrm{CH}}_{3}$ ) group or the two protons of a methylene ( ${\mathrm{CH}}_{2}$ ) group. Therefore, for each external nucleus $m\notin G_{P}$ , the J coupling between m and any spin $p\in G_{P}$ is the same: $J^{mp}=J^{mG_{P}}$ . Thus, the J -coupling constant is independent of the index of the nucleus within $G_{P}$ and depends only on the outer nucleus m . The magnetic equivalence enables us to treat the group $G_{P}$ as a pseudo-nucleus with total spin K . The Hilbert space of P identical spin- 1/2 nuclei can be decomposed into invariant subspaces corresponding to definite total spin (Messiah, 1962):

2 $$\overset{P}{\underset{i=1}{⊗}}D_{1/2}^{i}=\overset{P/2}{\underset{K=K_{min}}{⊕}}\mu_{K}D_{K},$$

where $D_{1/2}^{i}$ denotes the spin- 1/2 representation for spin i , $D_{K}$ is the irreducible representation of the collective spin system corresponding to total spin K , and

3 μK=2K+1P/2+K+1PP/2-K

is the multiplicity of spin K , where nk=n!/[(n-k)!k!] is the binomial coefficient. This commutation relation, $[{\hat{K}}^{2},\hat{H}(t)]=0$ , allows the Hamiltonian in Eq. (1) to be block diagonalized according to the total spin K of the group $G_{P}$ (Barskiy and Pravdivtsev, 2025):

4 $$\hat{H}(t)=\overset{P/2}{\underset{K=K_{min}}{⊕}}\langle K|\hat{H}(t)|K\rangle =\overset{P/2}{\underset{K=K_{min}}{⊕}}{\hat{H}}_{K}(t),$$

where

5 $$K_{min}=\left\{\begin{array}{ll} 0 & \text{if }P\text{ is even},\\ 1/2 & \text{if }P\text{ is odd}. \end{array}\right.$$

Similarly, when the density matrix of the full spin ensemble commutes with the total spin operator, $[{\hat{K}}^{2},\hat{\rho }(t)]=0$ , it can be decomposed into the corresponding invariant subspaces:

6 $$\hat{\rho }(t)=\overset{P/2}{\underset{K=K_{min}}{⊕}}g_{K}{\hat{\rho }}_{K}(t),$$

where

7 $$g_{K}=\frac{(2K+1)\mu_{K}}{2^{P}}$$

is a statistical weight of the K subspace with $\sum_{K=K_{min}}^{P/2}g_{K}=1$ . Each block of the density matrix is normalized in the usual way, $\text{Tr}\{{\hat{\rho }}_{K}(t)\}=1$ . For an observable $\hat{O}$ that commutes with the total spin operator, $[{\hat{K}}^{2},\hat{O}]=0$ , the expectation value can be calculated block-wise. First, the operator is decomposed into the following corresponding blocks:

8 $$\hat{O}=\overset{P/2}{\underset{K=K_{min}}{⊕}}{\hat{O}}_{K},$$

and then the ensemble average is obtained as

9 $$\begin{array}{rl} \langle \hat{O}\rangle (t) & =\text{Tr}\{\hat{O}\hat{\rho }(t)\}=\sum_{K=K_{min}}^{P/2}g_{K}\;\text{Tr}\{{\hat{O}}_{K}{\hat{\rho }}_{K}(t)\}\\ & =\sum_{K=K_{min}}^{P/2}g_{K}\;\langle {\hat{O}}_{K}\rangle (t). \end{array}$$

#### 2.1.2 The z projection of total spin

Each block ${\hat{H}}_{K}(t)$ corresponding to the total spin K of the group $G_{P}$ can be further decomposed according to the z projection of the total spin. Specifically, consider a single K block:

10 $$\begin{array}{rl} {\hat{H}}_{K}(t) & =-B_{0}(t)\left(\sum_{l=1}^{N-P}\gamma^{l}{\hat{I}}_{z}^{l}+\gamma^{P}{\hat{K}}_{z}\right)\\ & +2\pi \sum_{l<m}^{N-P}J^{lm}({\hat{I}}^{l}\cdot {\hat{I}}^{m})+2\pi \sum_{l=1}^{N-P}J^{lG_{P}}({\hat{I}}^{l}\cdot \hat{K}), \end{array}$$

where $\gamma^{P}$ is the gyromagnetic ratio of a nucleus in the group $G_{P}$ of P magnetically equivalent nuclei, and $J^{lG_{P}}$ denotes the J -coupling constant between an external nucleus $l\notin G_{P}$ and the nuclei within the group $G_{P}$ . J couplings within the group $G_{P}$ are omitted as they do not affect the dynamics within the K block. The Hamiltonian ${\hat{H}}_{K}(t)$ commutes with the z projection of the total spin of all nuclei, $[{\hat{F}}_{z},{\hat{H}}_{K}(t)]=0$ , where

11 $${\hat{F}}_{z}=\sum_{l=1}^{N-P}{\hat{I}}_{z}^{l}+{\hat{K}}_{z},$$

and the first sum runs over a set of non-equivalent spin- 1/2 nuclei outside the group $G_{P}$ . Consequently, ${\hat{H}}_{K}(t)$ is block diagonal with respect to the eigenvalues of ${\hat{F}}_{z}$ , which takes values $F_{z}\in \left\{\frac{N-P}{2}+K,\frac{N-P}{2}+K-1,\ldots ,-\frac{N-P}{2}-K\right\}$ . In other words, ${\hat{H}}_{K}(t)$ can be decomposed as (2K+1+N-P) blocks:

12 $$\begin{array}{rl} {\hat{H}}_{K}(t) & =\overset{(N-P)/2+K}{\underset{F_{z}=-(N-P)/2-K}{⊕}}\langle F_{z}|{\hat{H}}_{K}(t)|F_{z}\rangle \\ & =\overset{(N-P)/2+K}{\underset{F_{z}=-(N-P)/2-K}{⊕}}{\hat{H}}_{K,F_{z}}(t), \end{array}$$

with each block ${\hat{H}}_{K,F_{z}}(t)$ corresponding to specific values of K and $F_{z}$ .

#### 2.1.3 Coherent spin dynamics

The time evolution of the density matrix $\hat{\rho }(t)$ is governed by the Liouville–von Neumann (LvN) equation:

13 $$\frac{\partial \hat{\rho }(t)}{\partial t}=-i[\hat{H}(t),\hat{\rho }(t)],$$

with an initial state $\hat{\rho }(0)$ . If the initial state commutes with the symmetry operators, $[{\hat{K}}^{2},\hat{\rho }(0)]=[{\hat{F}}_{z},\hat{\rho }(0)]=0$ , then the block-diagonal decomposition of $\hat{\rho }(0)$ is valid:

14 $$\begin{array}{rl} \hat{\rho }(0) & =\overset{P/2}{\underset{K=K_{min}}{⊕}}g_{K}{\hat{\rho }}_{K}(0)\\ & =\overset{P/2}{\underset{K=K_{min}}{⊕}}\overset{(N-P)/2+K}{\underset{F_{z}=-(N-P)/2-K}{⊕}}g_{K}{\hat{\rho }}_{K,F_{z}}(0), \end{array}$$

where we introduced the reduced density matrices ${\hat{\rho }}_{K,F_{z}}(t)$ . The dimension of the $F_{z}$ block in the Hilbert space H , denoted as $\text{dim}(H_{F_{z}})$ , is given by

15 dim(HFz)=∑mK=-KKN-PFz+(N-P)/2-mK.

Moreover, if the Hamiltonian commutes with the same symmetry operators,

16 $$\begin{array}{rl} \text{If }[{\hat{K}}^{2}, & \hat{H}(t)]=[{\hat{F}}_{z},\hat{H}(t)]=0,\\ \text{then } & [{\hat{K}}^{2},\hat{\rho }(t)]=[{\hat{F}}_{z},\hat{\rho }(t)]=0\text{ for any }t. \end{array}$$

This means that the block-diagonal structure of the density matrix is conserved for all instants of time:

17 $$\hat{\rho }(t)=\overset{P/2}{\underset{K=K_{min}}{⊕}}\overset{(N-P)/2+K}{\underset{F_{z}=-(N-P)/2-K}{⊕}}g_{K}{\hat{\rho }}_{K,F_{z}}(t)\text{ for any }t.$$

The reduced density matrices ${\hat{\rho }}_{K,F_{z}}(t)$ obey the LvN equation independently within each block:

18 $$\frac{\partial {\hat{\rho }}_{K,F_{z}}(t)}{\partial t}=-i[{\hat{H}}_{K,F_{z}}(t),{\hat{\rho }}_{K,F_{z}}(t)].$$

Thus, the block-diagonal decomposition reduces the computational complexity by considering smaller blocks with fixed values of K and $F_{z}$ independently.

### 2.2 Block-diagonal decomposition of Liouville space

#### 2.2.1 Coherent spin dynamics

In Liouville space, an operator $\hat{A}$ from Hilbert space is mapped to a commutation superoperator $\hat{\hat{A}}$ acting on an operator $\hat{O}$ according to

19 $$\hat{\hat{A}}\;\hat{O}=[\hat{A},\hat{O}].$$

**Figure 2:**
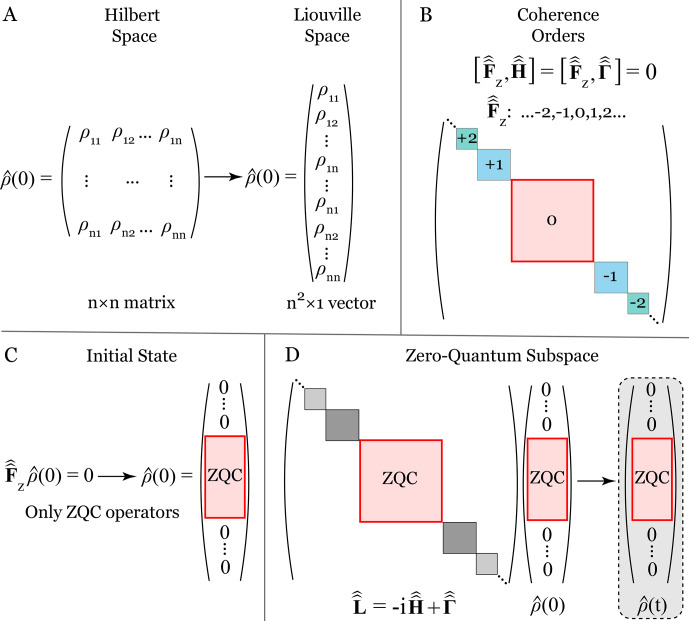
Schematic illustration of the zero-quantum coherence (ZQC) reduction in Liouville space. **(A)** Transformation of the density matrix $\hat{\rho }(t)$ from Hilbert space to Liouville space. **(B)** Decomposition of the Hamiltonian and the relaxation superoperators according to the coherence order, i.e., the eigenvalues of ${\hat{\hat{F}}}_{z}$ : + 2 (double-quantum coherence, + DQC), + 1 (single-quantum coherence, + SQC), 0 (zero-quantum coherence, ZQC), and the corresponding negative coherence orders. **(C)** The initial state of the system is assumed to satisfy ${\hat{\hat{F}}}_{z}\hat{\rho }(0)=0$ so that only ZQC operators are initially populated. **(D)** Only the ZQC subspace is relevant for spin evolution under the total Liouvillian $\hat{\hat{L}}=-i\hat{\hat{H}}+\hat{\hat{\Gamma }}$ , implying that ${\hat{\hat{F}}}_{z}\hat{\rho }(t)=0$ , and $\hat{\rho }(t)$ has no components outside the ZQC subspace. Consequently, all other coherence order subspaces can be omitted without affecting the spin dynamics.

At the same time, the density matrix in Liouville space is represented as a vector; see Fig. 2A. Throughout this paper, operators are denoted by hatted symbols, whereas superoperators are denoted by bold double-hatted symbols. Under this mapping, the Hamiltonian $\hat{H}(t)$ is represented by the Liouville space superoperator $\hat{\hat{H}}(t)$ , and similarly, the effective-spin operator ${\hat{K}}^{2}$ is mapped to the superoperator ${\hat{\hat{K}}}^{2}$ . In Liouville space, the coherent spin dynamics is described by the LvN equation:

20 $$\frac{\partial \hat{\rho }(t)}{\partial t}=-i\hat{\hat{H}}(t)\;\hat{\rho }(t).$$

If the initial density matrix commutes with the effective-spin operator ${\hat{K}}^{2}$ , i.e.,

21 $$\text{If }[{\hat{K}}^{2},\hat{\rho }(0)]=0,\text{ then }{\hat{\hat{K}}}^{2}\;\hat{\rho }(0)=0.$$

Moreover, Liouville space conserves commutation relations so that

22 $$\text{If }[{\hat{K}}^{2},\hat{H}(t)]=0,\text{ then }[{\hat{\hat{K}}}^{2},\hat{\hat{H}}(t)]=0.$$

Consequently, the block-diagonal structure of the density matrix is conserved during the evolution

23 $$\text{so that }{\hat{\hat{K}}}^{2}\;\hat{\rho }(t)=0,\text{ meaning }[{\hat{K}}^{2},\hat{\rho }(t)]=0.$$

Therefore, the blocks corresponding to different total spin values K remain uncoupled. Analogous to the Hilbert space treatment, in Liouville space we can also treat the group $G_{P}$ of P magnetically equivalent nuclear spins as a pseudo-nucleus with total spin $K\in \left\{\frac{P}{2},\frac{P}{2}-1,\ldots ,K_{min}\right\}.$

Consequently, the dynamics can be considered independently for each K subspace, and the expectation value of an observable is obtained by averaging over the blocks according to Eqs. (8) and (9), as in Hilbert space. The time evolution of the reduced density matrix ${\hat{\rho }}_{K}(t)$ in Liouville space is then given by

24 $$\frac{\partial {\hat{\rho }}_{K}(t)}{\partial t}=-i{\hat{\hat{H}}}_{K}(t)\;{\hat{\rho }}_{K}(t),$$

where we introduced the Hamiltonian superoperator reduced onto the K subspace as ${\hat{\hat{H}}}_{K}(t)\;•=[{\hat{H}}_{K}(t),•]$ , and the density matrix is normalized as $\text{Tr}\{{\hat{\rho }}_{K}(t)\}=1$ .

The symmetry associated with ${\hat{F}}_{z}$ in Hilbert space, see Eq. (11), is directly inherited in Liouville space. To analyze this, the operator ${\hat{F}}_{z}$ is mapped to the corresponding superoperator ${\hat{\hat{F}}}_{z}$ using Eq. (19). The eigenvalues of ${\hat{\hat{F}}}_{z}$ are commonly referred to as the *coherence orders*, which are well-known in standard high-field NMR; see Appendix A for details. A key property of the Liouville space evolution of Eq. (24) is

25 $$\begin{array}{rl} \text{If }{\hat{\hat{F}}}_{z}\;{\hat{\rho }}_{K}(0) & =0\text{ and }[{\hat{\hat{F}}}_{z},{\hat{\hat{H}}}_{K}(t)]=0,\\ & \text{then }{\hat{\hat{F}}}_{z}\;{\hat{\rho }}_{K}(t)=0\text{ for any }t. \end{array}$$

Equation (25) is schematically illustrated in Fig. 2B–D. In other words, the coherent dynamics in Liouville space conserves evolution strictly within the subspace of zero eigenvalue of ${\hat{\hat{F}}}_{z}$ , i.e., the zero-quantum coherence (ZQC) block. The ZQC subspace is spanned by operators of the following form, as discussed in Appendix A:

26 $$\text{ZQC}=\text{span}\{|F_{z},\xi \rangle \langle F_{z},\lambda |\},$$

with $F_{z}\in \left\{\frac{N-P}{2}+K,\frac{N-P}{2}+K-1,\ldots ,-\frac{N-P}{2}-K\right\}$ and ξ,λ run over all possible magnetic quantum numbers of the individual nuclear spins for a given value of the z projection of total spin $F_{z}$ .

The coherent evolution within the ZQC block is then governed by the standard LvN equation; see Eq. (24):

27 $$\frac{\partial {\hat{\rho }}_{K}(t)}{\partial t}=-i{\hat{\hat{H}}}_{K}^{\text{ZQC}}(t)\;{\hat{\rho }}_{K}(t),$$

where the dynamics is restricted to the ZQC subspace, as illustrated in Fig. 2D. Here, we also introduced ${\hat{\hat{H}}}_{K}^{\text{ZQC}}(t)$ as the Hamiltonian superoperator projected onto the ZQC subspace. The subscript ZQC of the density matrix is omitted since ${\hat{\rho }}_{K}(t)\equiv 0$ outside the ZQC subspace; see Fig. 2D.

We also emphasize that the consideration of the ZQC subspace drastically reduces the dimensionality of the problem. The full Liouville space has a dimension of $4^{N}$ , whereas the ZQC subspace with the effective-spin reduction has a smaller dimension:

28 dim(ZQC)=∑Fz=-(N-P)/2-K(N-P)/2+Kdim(HFz)2=∑Fz=-(N-P)/2-K(N-P)/2+K∑mK=-KKN-PFz+(N-P)/2-mK2<4N,

where Eq. (15) was used to calculate $\text{dim}(H_{F_{z}})$ . This restriction to the ZQC subspace allows efficient treatment of spin dynamics by working with a smaller independent block rather than the full Liouville space.

#### 2.2.2 Relaxational spin dynamics

Relaxational spin dynamics is described within the Redfield formalism, which leads to the following equation of motion for the density matrix $\hat{\rho }(t)$ :

29 $$\frac{\partial \hat{\rho }(t)}{\partial t}=(-i\;\hat{\hat{H}}(t)+\hat{\hat{\Gamma }})\hat{\rho }(t)=\hat{\hat{L}}(t)\;\hat{\rho }(t),$$

where $\hat{\hat{H}}(t)$ is the coherent Hamiltonian superoperator, $\hat{\hat{\Gamma }}$ is the relaxational superoperator describing stochastically modulated dynamics, and $\hat{\hat{L}}(t)=-i\;\hat{\hat{H}}(t)+\hat{\hat{\Gamma }}$ is the total Liouvillian. In this work, we used the random fluctuating field (RFF) mechanism in the fast-motion limit, also known as the extreme narrowing regime. Within this approximation, the relaxation superoperator takes the following form (Pileio, 2010):

30 $$\hat{\hat{\Gamma }}=-\frac{1}{2}\sum_{n,l}^{N}\frac{C^{nl}}{\sqrt{T_{1}^{n}T_{1}^{l}}}\sum_{m=-1}^{1}(-1{)}^{m}{\hat{\hat{T}}}_{1,-m}^{n}\;{\hat{\hat{T}}}_{1,m}^{l},$$

where we assumed that the local fields are isotropic and introduced the following quantities: $T_{1}^{n}$ is the longitudinal $T_{1}$ -relaxation time of the nucleus n , $C^{nl}\in [0,1]$ is a correlation constant between the local fluctuating magnetic fields at the spatial positions of nuclear spins n and l (Freeman et al., 1970), and ${\hat{\hat{T}}}_{1,m}^{n}\;•=[{\hat{T}}_{1,m}^{n},•]$ is the rank-1 irreducible spherical superoperator acting on nucleus n . The corresponding spherical tensor operators are defined as

31 $${\hat{T}}_{1,1}^{n}=-\frac{{\hat{I}}_{x}^{n}+i{\hat{I}}_{y}^{n}}{\sqrt{2}},{\hat{T}}_{1,0}^{n}={\hat{I}}_{z}^{n},{\hat{T}}_{1,-1}^{n}=\frac{{\hat{I}}_{x}^{n}-i{\hat{I}}_{y}^{n}}{\sqrt{2}}.$$

For the spin systems containing a group $G_{P}$ of P magnetically equivalent nuclei (e.g. ${\mathrm{CH}}_{2}$ or ${\mathrm{CH}}_{3}$ groups), Eq. (30) can be rewritten in a more convenient form by separating contributions from equivalent and non-equivalent spins:

32 $$\begin{array}{rl} \hat{\hat{\Gamma }} & =-\frac{1}{2}\sum_{n\notin G_{P}}\frac{1}{T_{1}^{n}}\sum_{m=-1}^{1}(-1{)}^{m}\;{\hat{\hat{T}}}_{1,-m}^{n}\;{\hat{\hat{T}}}_{1,m}^{l}\\ & -\frac{1}{2T_{1}^{P}}\sum_{n,l\in G_{P}}\sum_{m=-1}^{1}(-1{)}^{m}\;{\hat{\hat{T}}}_{1,-m}^{n}\;{\hat{\hat{T}}}_{1,m}^{l}, \end{array}$$

where we have explicitly accounted for the equivalence of the nuclei in group $G_{P}$ . Specifically, all nuclei in $G_{P}$ are assumed to have identical longitudinal relaxation times $T_{1}^{P}$ , and the local fluctuating magnetic fields at their spatial positions are taken to be fully correlated, such that $C^{nl}=1$ for $n,l\in G_{P}$ . All other local fields are assumed to be uncorrelated; i.e., $C^{nl}=0$ if either $n\notin G_{P}$ or $l\notin G_{P}$ .

The second term in Eq. (32), and therefore the full relaxation superoperator $\hat{\hat{\Gamma }}$ , commutes with the superoperator of the total spin of the group $G_{P}$ , ${\hat{\hat{K}}}^{2}$ . This follows from the identity

33 $$\sum_{n,l\in G_{P}}\sum_{m=-1}^{1}(-1{)}^{m}\;{\hat{\hat{T}}}_{1,-m}^{n}\;{\hat{\hat{T}}}_{1,m}^{l}=\sum_{n,l\in G_{P}}({\hat{\hat{T}}}_{1}^{n}\cdot {\hat{\hat{T}}}_{1}^{l})\;,$$

which represents a scalar product of rank-1 spherical superoperators in Liouville space (Pyper, 1971). This is fully analogous to the Hilbert space, where the scalar product of equivalent nuclear spins, $\sum_{n,l\in G_{P}}({\hat{I}}^{\;n}\cdot {\hat{I}}^{\;l})$ , commutes with ${\hat{K}}^{2}$ . As a consequence, $[{\hat{\hat{K}}}^{2},\hat{\hat{\Gamma }}]=0$ , and the relaxation superoperator can be decomposed into independent blocks corresponding to different values of ${\hat{\hat{K}}}^{2}$ . Importantly, we are interested only in the zero-eigenvalue subspace of ${\hat{\hat{K}}}^{2}$ since

34 $$\begin{array}{rl} \text{If }{\hat{\hat{K}}}^{2}\;\hat{\rho }(0)=0\text{ and } & [{\hat{\hat{K}}}^{2},\hat{\hat{\Gamma }}]=[{\hat{\hat{K}}}^{2},\hat{\hat{H}}(t)]=0,\\ & \text{then }{\hat{\hat{K}}}^{2}\;\hat{\rho }(t)=0\text{ for any }t. \end{array}$$

Therefore, relaxational dynamics also conserves the density matrix $\hat{\rho }(t)$ within the zero-eigenvalue subspace of ${\hat{\hat{K}}}^{2}$ ; i.e. ${\hat{\hat{K}}}^{2}\;\hat{\rho }(t)=[{\hat{K}}^{2},\hat{\rho }(t)]=0$ . As a consequence, the blocks of the density matrix corresponding to different values of the total spin K of the magnetically equivalent nuclei in the group $G_{P}$ are not mixed by relaxation. This allows us to treat relaxation independently for each value of the nuclear spin $K\in \left\{\frac{P}{2},\frac{P}{2}-1,\ldots ,K_{min}\right\}$ of the group $G_{P}$ (Doronin et al., 2011) and introduce the reduced relaxation superoperator

35 $$\begin{array}{rl} {\hat{\hat{\Gamma }}}_{K} & =-\frac{1}{2}\sum_{n\notin G_{P}}\frac{1}{T_{1}^{n}}\sum_{m=-1}^{1}(-1{)}^{m}\;{\hat{\hat{T}}}_{1,-m}^{n}\;{\hat{\hat{T}}}_{1,m}^{n}\\ & -\frac{1}{2T_{1}^{P}}\sum_{m=-1}^{1}(-1{)}^{m}\;{\hat{\hat{T}}}_{1(K),-m}\;{\hat{\hat{T}}}_{1(K),m}, \end{array}$$

where ${\hat{\hat{T}}}_{1(K),m}$ is a rank-1 irreducible spherical superoperator constructed for a spin K according to Eq. (31). A crucial property of the superoperator ${\hat{\hat{\Gamma }}}_{K}$ is that it commutes with the superoperator ${\hat{\hat{F}}}_{z}$ :

36 $$\left[{\hat{\hat{F}}}_{z},{\hat{\hat{\Gamma }}}_{K}\right]=0.$$

This follows from the identities

37 $$\begin{array}{rl} & \sum_{m=-1}^{1}(-1{)}^{m}\;{\hat{\hat{T}}}_{1,-m}^{n}\;{\hat{\hat{T}}}_{1,m}^{n}=\left({\hat{\hat{T}}}_{1}^{n}\cdot {\hat{\hat{T}}}_{1}^{n}\right),\\ & \sum_{m=-1}^{1}(-1{)}^{m}\;{\hat{\hat{T}}}_{1(K),-m}\;{\hat{\hat{T}}}_{1(K),m}=\left({\hat{\hat{T}}}_{1(K)}\cdot {\hat{\hat{T}}}_{1(K)}\right), \end{array}$$

which represent scalar products of rank-1 spherical superoperators in Liouville space. Such scalar products are rotationally invariant and therefore commute with ${\hat{\hat{F}}}_{z}$ (this can also be verified by direct evaluation of the commutators). Combining Eqs. (25) and (36), we obtain ${\hat{\hat{F}}}_{z}\;{\hat{\rho }}_{K}(t)=0$ for any t .

Thus, relaxational dynamics driven by ${\hat{\hat{\Gamma }}}_{K}$ and, therefore, the full Liouville space dynamics governed by Eq. (29) conserve the evolution within the ZQC subspace; see Fig. 2B–D. Therefore, the evolution equation takes the following form:

38 $$\begin{array}{rl} \frac{\partial {\hat{\rho }}_{K}(t)}{\partial t} & =(-i\;{\hat{\hat{H}}}_{K}^{\text{ZQC}}(t)+{\hat{\hat{\Gamma }}}_{K}^{\text{ZQC}})\hat{\rho }(t)\\ & ={\hat{\hat{L}}}_{K}^{\text{ZQC}}(t)\;\hat{\rho }(t), \end{array}$$

where we introduced ${\hat{\hat{L}}}_{K}^{\text{ZQC}}(t)$ as the Liouvillian superoperator projected onto the ZQC subspace. We emphasize that all components of the density matrix outside the ZQC subspace are identically zero, as shown in Fig. 2C and D.

It should be noted that a wide class of relaxation mechanisms possesses the same symmetry properties: the effective-spin reduction and conservation of a coherence order (Kowalewski and Mäler, 2006):

39 $$[{\hat{\hat{K}}}^{2},\hat{\hat{\Gamma }}]=\left[{\hat{\hat{F}}}_{z},\hat{\hat{\Gamma }}\right]=0,$$

including dipole–dipole relaxation, chemical shift anisotropy (CSA) with axial symmetry, and cross-correlated relaxation between these mechanisms. Nevertheless, for the majority of SABRE experiments, the random fluctuating field (RFF) model provides a sufficiently accurate description of relaxation.

### 2.3 Block-diagonal decomposition of SABRE master equations

**Figure 3:**
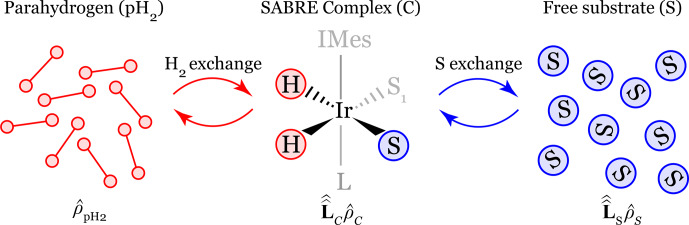
Schematic representation of the SABRE polarization transfer process from parahydrogen (pH_2_) to the substrate (S) mediated by the transient polarization transfer complex (C). Spin dynamics within the complex is governed by the Liouvillian superoperator ${\hat{\hat{L}}}_{C}$ , while spin evolution in the pool of free substrate molecules is described by ${\hat{\hat{L}}}_{S}$ . In our model, parahydrogen supply and exchange with the complex are assumed to be fast such that the overall exchange is effectively determined by the substrate dissociation rate constant $k_{d}$ and association rate $W_{a}$ . Here, L and S_1_ denote the axial and equatorial ligand of the complex, respectively, and IMes is a heterocyclic carbene ligand (1,3-bis(2,4,6-trimethylphenyl)-1,3-dihydro-2H-imidazol-2-ylidene).

The SABRE hyperpolarization process arises from an intricate interplay between spin and chemical exchange dynamics. Polarization is transferred from a parahydrogen molecule to the substrate during its transient binding within the Ir-based polarization transfer complex, as illustrated in Fig. 3. Inside the complex, the nuclear spins of the parahydrogen-derived hydrides and the nuclear spins of the substrate become strongly coupled, leading to the formation of entangled spin states. This coherent spin evolution enables efficient transfer of spin order from parahydrogen to the substrate nuclei. Upon dissociation of the substrate from the metal complex, the accumulated spin polarization is released into the free substrate, resulting in its observable hyperpolarization of the nuclear spins.

Thus, spin dynamics governs the polarization transfer processes occurring within the metal complex, whereas chemical dynamics determines how this polarization is transferred to and accumulated in the ensemble of free substrate molecules. Both coherent spin evolution and stochastic chemical exchange are therefore essential ingredients of the SABRE mechanism.

To rigorously consider this sophisticated SABRE dynamics, we used the SABRE master equation (Knecht et al., 2016):

40 $$\left\{\begin{array}{rl} \frac{d{\hat{\rho }}_{S}(t)}{dt} & ={\hat{\hat{L}}}_{S}(t)\;{\hat{\rho }}_{S}(t)-W_{a}\;{\hat{\rho }}_{S}(t)+k_{d}\;{\text{Tr}}_{H_{2}}\{{\hat{\rho }}_{C}(t)\},\\ \frac{d{\hat{\rho }}_{C}(t)}{dt} & ={\hat{\hat{L}}}_{C}(t)\;{\hat{\rho }}_{C}(t)-k_{d}\;{\hat{\rho }}_{C}(t)\\ & +W_{a}\;\{{\hat{\rho }}_{S}(t)⊗{\hat{\rho }}_{{\text{pH}}_{2}}\}, \end{array}\right.$$

where we introduced the following quantities: ${\hat{\rho }}_{S,C}(t)$ values are the density matrices of the free substrate and the SABRE complex, respectively; ${\hat{\rho }}_{{\text{pH}}_{2}}$ is the density matrix of parahydrogen (singlet spin state of two nuclei); ${\hat{\hat{L}}}_{S,C}(t)=-i{\hat{\hat{H}}}_{S,C}(t)+{\hat{\hat{\Gamma }}}_{S,C}$ values are the Liouvillian superoperators of the substrate and the complex; $k_{d}$ is the substrate dissociation rate constant; and $W_{a}=k_{d}[C]/[S]$ is the substrate association rate constant. Importantly, in Eq. (40) the density matrices are normalized by the following concentrations: $\text{Tr}\{{\hat{\rho }}_{S}(t)\}=[S]/([C]+[S])$ and $\text{Tr}\{{\hat{\rho }}_{C}(t)\}=[C]/([C]+[S])$ . Equation (40) arises as the rigorous Markovian limit of the integral encounter theory, a first-principle approach to chemical kinetics in the condensed phase (Doktorov et al., 2002; Feskov et al., 2005; Ivanov et al., 2004). SABRE Eq. (40) is linear and valid in the regime of fast parahydrogen supply and fast dihydrogen exchange with the complex. Although the full SABRE kinetics is generally non-linear (Pravdivtsev and Hövener, 2019), the linear approximation is adequate for most practical purposes.

Equation (40) can be transformed to the superoperator form

41 $$\left\{\begin{array}{rl} \frac{d{\hat{\rho }}_{S}(t)}{dt} & ={\hat{\hat{A}}}_{S}(t)\;{\hat{\rho }}_{S}(t)+k_{d}\;{\hat{\hat{S}}}_{{\text{TrH}}_{2}}\;{\hat{\rho }}_{C}(t),\\ \frac{d{\hat{\rho }}_{C}(t)}{dt} & ={\hat{\hat{A}}}_{C}(t)\;{\hat{\rho }}_{C}(t)+W_{a}\;{\hat{\hat{S}}}_{\text{Kron}}\;{\hat{\rho }}_{S}(t). \end{array}\right.$$

Here, we introduce the following auxiliary superoperators:

42 $$\begin{array}{rl} {\hat{\hat{A}}}_{S}(t) & ={\hat{\hat{L}}}_{S}(t)-W_{a}\;{\hat{\hat{1}}}_{S},\\ {\hat{\hat{A}}}_{C}(t) & ={\hat{\hat{L}}}_{C}(t)-k_{d}\;{\hat{\hat{1}}}_{C}, \end{array}$$

where ${\hat{\hat{1}}}_{S,C}$ values are the identity superoperators acting on the density matrices according to ${\hat{\hat{1}}}_{S}{\hat{\rho }}_{S}(t)={\hat{\rho }}_{S}(t)$ and ${\hat{\hat{1}}}_{C}{\hat{\rho }}_{C}(t)={\hat{\rho }}_{C}(t)$ , ${\hat{\hat{S}}}_{{\text{TrH}}_{2}}\;{\hat{\rho }}_{C}(t)={\text{Tr}}_{H_{2}}\{{\hat{\rho }}_{C}(t)\}$ is the partial trace superoperator over the states of the hydrides in the complex, and ${\hat{\hat{S}}}_{\text{Kron}}\;{\hat{\rho }}_{S}(t)=\{{\hat{\rho }}_{S}(t)⊗{\hat{\rho }}_{{\text{pH}}_{2}}\}$ is the Kronecker product superoperator.

The initial state of the complex and the free substrate is typically assumed to be completely non-polarized:

43 $$\begin{array}{rl} {\hat{\rho }}_{S}(0) & =\frac{\left[S\right]}{\left[C]+[S\right]}\frac{{\hat{1}}_{S}}{\text{dim}(H_{S})},\\ {\hat{\rho }}_{C}(0) & =\frac{\left[C\right]}{\left[C]+[S\right]}\frac{{\hat{1}}_{C}}{\text{dim}(H_{C})}, \end{array}$$

where ${\hat{1}}_{S,C}$ values are the identity operators, and $\text{dim}(H_{S,C})$ values are the dimensions of the Hilbert spaces of the free substrate and the complex, respectively.

#### 2.3.1 The group of magnetically equivalent nuclei

In what follows, we demonstrate how block-diagonal decomposition can be employed to reduce the dimensionality of Eq. (41).

We note that the initial density matrices in Eq. (43) commute with any operator in the Hilbert space, as they are proportional to the identity operator. Consequently, if the substrate contains a group of magnetically equivalent nuclei $G_{P}$ , it can be treated as a single effective nucleus with total spin K ; see Eqs. (4) and (5). This reduction is justified because Eq. (34) holds for both the free substrate and the SABRE complex. In this case, where the magnetic equivalence is conserved in the free substrate and in the complex, the chemical exchange superoperators do not mix subspaces with different values of K .

As a consequence, the first step in the dimensionality reduction is to solve Eq. (41) independently for each value of the total spin K of the group of magnetically equivalent nuclei. To avoid unnecessary notational complexity, the index K will be omitted in what follows, with the understanding that the density matrices ${\hat{\rho }}_{S}(t)$ and ${\hat{\rho }}_{C}(t)$ correspond to a fixed value of K .

#### 2.3.2 The z projection of total spin

The next step is to exploit the symmetry associated with the z projection of the total spin of the substrate, ${\hat{\hat{F}}}_{Sz}$ , and of the complex, ${\hat{\hat{F}}}_{Cz}$ . In particular, the following relations hold:

44 $$\left\{\begin{array}{rl} {\hat{\hat{F}}}_{Sz}\;{\hat{\rho }}_{S}(0) & =0,\\ \left[{\hat{\hat{F}}}_{Sz},{\hat{\hat{A}}}_{S}\right] & =0. \end{array}\right.\left\{\begin{array}{rl} {\hat{\hat{F}}}_{Cz}\;{\hat{\rho }}_{C}(0) & =0,\\ \left[{\hat{\hat{F}}}_{Cz},{\hat{\hat{A}}}_{C}\right] & =0. \end{array}\right.$$

The last two identities follow from the fact that the relaxation and coherent dynamics conserve the coherence order:

45 $$\begin{array}{rl} & \left[{\hat{\hat{F}}}_{Sz},{\hat{\hat{L}}}_{S}\right]=-i\underset{0}{\underset{︸}{\left[{\hat{\hat{F}}}_{Sz},{\hat{\hat{H}}}_{S}\right]}}+\underset{0}{\underset{︸}{\left[{\hat{\hat{F}}}_{Sz},{\hat{\hat{\Gamma }}}_{S}\right]}}=0,\\ & \left[{\hat{\hat{F}}}_{Cz},{\hat{\hat{L}}}_{C}\right]=-i\underset{0}{\underset{︸}{\left[{\hat{\hat{F}}}_{Cz},{\hat{\hat{H}}}_{C}\right]}}+\underset{0}{\underset{︸}{\left[{\hat{\hat{F}}}_{Cz},{\hat{\hat{\Gamma }}}_{C}\right]}}=0 \end{array}$$

and the definition of the auxiliary superoperators; see Eq. (42) ( ${\hat{\hat{1}}}_{S,C}$ commutes with any superoperator). As a result, the first terms in Eq. (41), governed by the auxiliary superoperators ${\hat{\hat{A}}}_{S,C}$ , conserve the dynamics within the zero-quantum coherence (ZQC) subspaces of the Liouville spaces of the complex and the free substrate. These subspaces are denoted as ${\text{ZQC}}_{C}$ and ${\text{ZQC}}_{S}$ , respectively.

Additionally, in SABRE the situation is significantly complicated by the presence of reversible chemical exchange. Special attention must therefore be paid to the exchange superoperators ${\hat{\hat{S}}}_{{\text{TrH}}_{2}}$ and ${\hat{\hat{S}}}_{\text{Kron}}$ , introduced in Eq. (41), since they explicitly couple the Liouville spaces of the complex and the free substrate. In Appendices B and C, we demonstrate that these chemical exchange superoperators conserve the zero coherence order. Consequently, ${\hat{\hat{S}}}_{\text{Kron}}:{\text{ZQC}}_{S}\to {\text{ZQC}}_{C},{\hat{\hat{S}}}_{{\text{TrH}}_{2}}:{\text{ZQC}}_{C}\to {\text{ZQC}}_{S}$ , and the following relations hold:

46 $${\hat{\hat{F}}}_{Sz}\;{\hat{\rho }}_{S}(t)=0\text{ and }{\hat{\hat{F}}}_{Cz}\;{\hat{\rho }}_{C}(t)=0\text{ for any }t.$$

**Figure 4:**
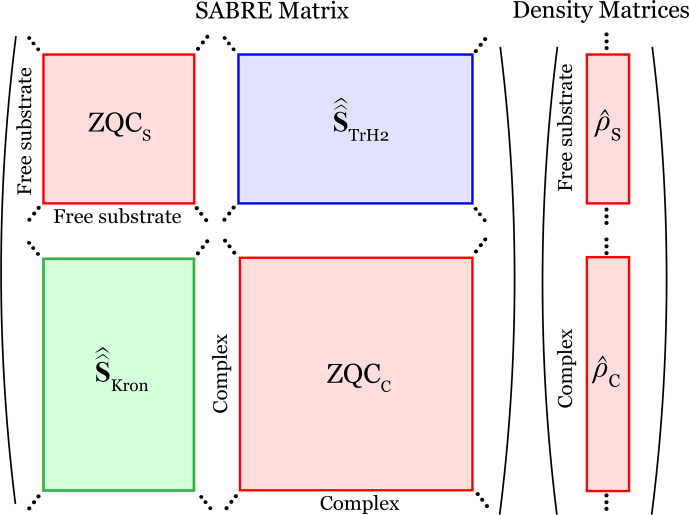
Block-diagonal decomposition for SABRE master equations. Only zero-quantum coherence (ZQC) subspaces of the free substrate ( ${\text{ZQC}}_{S}$ ) and the complex ( ${\text{ZQC}}_{C}$ ) are relevant in the SABRE matrix. Chemical exchange superoperators ${\hat{\hat{S}}}_{{\text{TrH}}_{2}}$ and ${\hat{\hat{S}}}_{\text{Kron}}$ couple these ZQC subspaces. Outside the ZQC subspaces, the density matrices of the free substrate ${\hat{\rho }}_{S}$ and the complex ${\hat{\rho }}_{C}$ are zero. The density matrices are represented in Liouville space as vectors.

Thus, the dynamics can be rigorously restricted to the ${\text{ZQC}}_{S}$ and ${\text{ZQC}}_{C}$ subspaces, as illustrated in Fig. 4. Importantly, all other coherence order subspaces can be omitted without affecting the description of SABRE spin dynamics, since the density matrices are identically zero outside the ZQC subspaces (Fig. 4).

Thus, the dimensionality of the problem is significantly reduced by considering that Eq. (41) is projected onto the ZQC subspaces instead of the full Liouville spaces:

47 $$\left\{\begin{array}{rl} \frac{d{\hat{\rho }}_{S}(t)}{dt} & ={\hat{\hat{A}}}_{S}^{\text{ZQC}}(t)\;{\hat{\rho }}_{S}(t)+k_{d}\;{\hat{\hat{S}}}_{{\text{TrH}}_{2}}^{\text{ZQC}}\;{\hat{\rho }}_{C}(t),\\ \frac{d{\hat{\rho }}_{C}(t)}{dt} & ={\hat{\hat{A}}}_{C}^{\text{ZQC}}(t)\;{\hat{\rho }}_{C}(t)+W_{a}\;{\hat{\hat{S}}}_{\text{Kron}}^{\text{ZQC}}\;{\hat{\rho }}_{S}(t), \end{array}\right.$$

where ${\hat{\hat{A}}}_{S}^{\text{ZQC}}(t)$ and ${\hat{\hat{A}}}_{C}^{\text{ZQC}}(t)$ are the superoperators ${\hat{\hat{A}}}_{S}(t)$ and ${\hat{\hat{A}}}_{C}(t)$ reduced onto the ${\text{ZQC}}_{S}$ and ${\text{ZQC}}_{C}$ subspaces, respectively, and ${\hat{\hat{S}}}_{{\text{TrH}}_{2}}^{\text{ZQC}}$ and ${\hat{\hat{S}}}_{\text{Kron}}^{\text{ZQC}}$ are the exchange superoperators calculated between the corresponding ZQC blocks. Importantly, these equations provide a rigorous, complete description of the full SABRE dynamics without any additional approximations while fully exploiting the block-diagonal structure of the density matrices to reduce computational complexity. In our notation, the ZQC subscript for the density matrices in Eq. (47) is omitted, as they are identically zero outside the zero-quantum coherence subspaces.

#### 2.3.3 The dimension of the SABRE matrix

In this work, we introduce three different SABRE matrices for comparison: the full SABRE matrix (without any reduction), the K-SABRE matrix (with effective-spin reduction for the magnetically equivalent nuclei), and the ZQC SABRE matrix (with both effective-spin and ZQC reductions). The dimensions of the matrices are simply the sum of the dimensions of the Liouville spaces for the free substrate and the complex:

48 $$\begin{array}{rl} & \text{dim}(\text{Full SABRE})=4^{N}+4^{N+2},\\ & \text{dim}(\text{K-SABRE})=(2K+1{)}^{2}\;(4^{N-P}+4^{N-P+2}), \end{array}$$

where N is the number of substrate spins, N+2 is the number of spins in the complex (see Fig. 4), N-P is the number of magnetically non-equivalent nuclear spins in the substrate, N-P+2 is the number of magnetically non-equivalent nuclear spins in the complex, and K is the total spin of the group $G_{P}$ of magnetically equivalent nuclei. In turn, the dimension of the ZQC SABRE matrix of Eq. (47) is given by the sum of dimensions of the ZQC subspaces:

49 dim(ZQC SABRE)=∑Fz=-(N-P)/2-K(N-P)/2+K∑mk=-KKN-PFz+(N-P)/2-mk2+∑Fz=-(N+2-P)/2-K(N+2-P)/2+K∑mk=-KKN+2-PFz+(N+2-P)/2-mk2,

where we used Eq. (28). The dimension of the ZQC-reduced SABRE matrix is significantly smaller than the dimension of the K-SABRE and the full SABRE matrices. The asymptotic behavior at $\sqrt{N-P}\gg K$ gives the following result (see Eq. 48 and Appendix D):

50 $$\begin{array}{rl} & \text{dim}(\text{Full SABRE})∼4^{N+2},\\ & \text{dim}(\text{K-SABRE})∼(2K+1{)}^{2}4^{N-P+2},\\ & \text{dim}(\text{ZQC SABRE})∼\frac{(2K+1{)}^{2}}{\sqrt{\pi (N-P)}}4^{N-P+2}. \end{array}$$

Thus, the K-SABRE matrix only uses reduction based on the effective-spin treatment, which allows us to reduce the dimension by the factor $\frac{4^{P}}{(2K+1{)}^{2}}$ . The following application of the ZQC reduction allows us to achieve better asymptotics for large spin systems since the dimension of the matrix is reduced by a greater factor of $\frac{\text{dim}(\text{Full SABRE})}{\text{dim}(\text{ZQC SABRE})}=\frac{\sqrt{\pi (N-P)}4^{P}}{(2K+1{)}^{2}}$ ≫ 1.

### 2.4 On the validity of the zero-quantum coherence reduction

Our framework with the ZQC reduction is valid if the following conditions are met.

1. **The Hamiltonian**
  The Hamiltonian of the spin system $\hat{H}(t)$ is assumed to commute with the z projection of the total spin ${\hat{F}}_{z}$ . This assumption holds as long as no transverse ( x or y ) radiofrequency (RF) pulses are applied. Consequently, the proposed framework is particularly valuable for low and ultralow fields, where nontrivial spin dynamics occurs “spontaneously”, i.e., in the absence of RF pulses. Specialized state-space reduction techniques are also employed in high-field NMR simulations, notably in the Spinach software framework, which enables large-scale simulations of spin dynamics under arbitrary RF pulse sequences (Kuprov et al., 2007; Kuprov, 2018; Hogben et al., 2011).
2. **The relaxation superoperator**
  The relaxation superoperator $\hat{\hat{\Gamma }}$ is assumed to commute with ${\hat{\hat{F}}}_{z}$ . This approximation is valid for a variety of relevant NMR relaxation mechanisms in the extreme narrowing regime, including dipolar relaxation, chemical shift anisotropy, and their cross-correlations. A possible exception is chemical shift anisotropy in the absence of axial symmetry, which represents a rather uncommon case.
3. **The initial condition**
  The initial density matrices of the free substrate, ${\hat{\rho }}_{S}(0)$ , and the SABRE complex, ${\hat{\rho }}_{C}(0)$ , are assumed to commute with their respective z projection of total spin operators; i.e., $[{\hat{F}}_{Sz},{\hat{\rho }}_{S}(0)]=0$ and $[{\hat{F}}_{Cz},{\hat{\rho }}_{C}(0)]=0$ . This condition is satisfied for the standard SABRE initial condition given by Eq. (43).

It should be noted that, if only relaxation and coherent evolution are considered without chemical exchange, the dynamics can be reduced onto subspaces with fixed magnetic quantum numbers $F_{C}$ and $F_{S}$ without invoking the full ZQC subspaces. Strictly speaking, Eq. (46) ensures that the density matrices ${\hat{\rho }}_{C}(t)$ and ${\hat{\rho }}_{S}(t)$ commute with ${\hat{F}}_{Cz}$ and ${\hat{F}}_{Sz}$ , implying their block-diagonal structure in $F_{C}$ and $F_{S}$ , respectively. However, the chemical exchange superoperator ${\hat{\hat{S}}}_{{\text{TrH}}_{2}}$ , which describes the decay of correlations between the substrate and hydrides in the complex, couples the subspaces of the free substrate and the complex with different quantum numbers. Consequently, although the density matrices remain block diagonal in magnetic quantum numbers $F_{C}$ and $F_{S}$ , the SABRE equations themselves are not block diagonal in $F_{C}$ and $F_{S}$ , meaning that they cannot be reduced to a single block with fixed $F_{C}$ and $F_{S}$ . Therefore, in SABRE, the full ZQC subspaces of the substrate and the complex must be considered.

## 3 Methods

### 3.1 The algorithm for numerical simulations

This section summarizes the theoretical framework and outlines the complete algorithm for numerical simulation.

1. **Identify permutation-equivalent spin groups.** Determine the group $G_{P}$ of magnetically equivalent spin- 1/2 nuclei within the substrate molecule. This typically includes protons in chemically equivalent groups, such as $-{\mathrm{CH}}_{2}-$ or $-{\mathrm{CH}}_{3}$ . If the molecule contains several distinct sets of magnetically equivalent nuclei, each is treated separately as groups $G_{P_{1}},G_{P_{2}},\ldots$ , containing $P_{1},P_{2},\ldots$ nuclei, respectively.
2. **Replace each equivalent group with an effective spin.** Each group $G_{P_{i}}$ of magnetically equivalent spins is replaced by a single effective spin $K_{i}$ , whose quantum number can take values in the range
51 $$K_{i}\in [K_{i,\text{min}},P_{i}/2],$$
  where $K_{i,\text{min}}=0$ if $P_{i}$ is even, and $K_{i,\text{min}}=1/2$ if $P_{i}$ is odd.
3. **Construct the effective spin system.** The original system of N nuclei is thereby reduced to a system consisting of the following:
  - the $\left(N-P_{1}-P_{2}-\ldots \right)$ non-equivalent spin- 1/2 nuclei,
  - a single effective spin $K_{1}$ (representing group $G_{P_{1}}$ ),
  - a single effective spin $K_{2}$ (representing group $G_{P_{2}}$ ), and so on.
4. **Build the Hamiltonian superoperators for the complex and free substrate.** The Hamiltonian operator for the effective-spin system reads as follows (see Eq. 10):
52 $$\begin{array}{rl} & \;{\hat{H}}_{K_{1},K_{2},\ldots }(t)=\\ & -B_{0}(t)\left(\sum_{l=1}^{N-P_{1}-P_{2}-\ldots }\gamma^{l}{\hat{I}}_{z}^{l}+\gamma^{P_{1}}{\hat{K}}_{1z}+\gamma^{P_{2}}{\hat{K}}_{2z}+\ldots \right)\\ & +2\pi \sum_{l<m}^{N-P_{1}-P_{2}-\ldots }J^{lm}({\hat{I}}^{l}\cdot {\hat{I}}^{m})\\ & +2\pi \sum_{l=1}^{N-P_{1}-P_{2}-\ldots }(J^{lG_{P_{1}}}({\hat{I}}^{l}\cdot {\hat{K}}_{1})+J^{lG_{P_{2}}}({\hat{I}}^{l}\cdot {\hat{K}}_{2})+\ldots )\\ & +2\pi (J^{G_{P_{1}}G_{P_{2}}}({\hat{K}}_{1}\cdot {\hat{K}}_{2})+J^{G_{P_{1}}G_{P_{3}}}({\hat{K}}_{1}\cdot {\hat{K}}_{3})+\ldots ), \end{array}$$
  where the sums run over all magnetically non-equivalent spin- 1/2 nuclei, and the remaining terms include the scalar J couplings within and between the different spin groups of the equivalent nuclei. The Hamiltonian is then mapped to Liouville space as the commutation superoperator; see Eq. (19):
53 $$\begin{array}{rl} \hat{\hat{H}} & {}_{K_{1},K_{2},\ldots }(t)=\\ & {\hat{H}}_{K_{1},K_{2},\ldots }(t)⊗\hat{1}-\hat{1}⊗{\hat{H}}_{K_{1},K_{2},\ldots }(t{)}^{⊤}, \end{array}$$
  where $\hat{1}$ is the identity operator, and ⊤ denotes the matrix transpose. The same construction is applied separately to the SABRE complex and the free substrate, using their respective spin systems and parameters.
5. **Build the relaxation superoperators for the complex and free substrate.** The relaxation superoperator for the effective-spin system is given by (see Eq. 35)
54 $$\begin{array}{rl} {\hat{\hat{\Gamma }}}_{K_{1},K_{2},\ldots } & =-\frac{1}{2}\sum_{n=1}^{N-P_{1}-P_{2}-\ldots }\frac{1}{T_{1}^{n}}\sum_{m=-1}^{1}(-1{)}^{m}\;{\hat{\hat{T}}}_{1,-m}^{n}{\hat{\hat{T}}}_{1,m}^{n}\\ & -\frac{1}{2T_{1}^{P_{1}}}\sum_{m=-1}^{1}(-1{)}^{m}\;{\hat{\hat{T}}}_{1(K_{1}),-m}{\hat{\hat{T}}}_{1(K_{1}),m}\\ & -\ldots , \end{array}$$
  where the first sum runs over all $\left(N-P_{1}-P_{2}-\ldots \right)$ magnetically non-equivalent spin- 1/2 nuclei, and other terms represent a contribution for a given group of magnetically equivalent spins with longitudinal relaxation time $T_{1}$ . The irreducible spherical superoperators are given by
55 $$\begin{array}{rl} & {\hat{\hat{T}}}_{1,m}^{n}={\hat{T}}_{1,m}^{n}⊗\hat{1}-\hat{1}⊗{\hat{T}}_{1,m}^{n⊤},\\ & {\hat{\hat{T}}}_{1(K_{i}),m}={\hat{T}}_{1(K_{i}),m}⊗\hat{1}-\hat{1}⊗{\hat{T}}_{1(K_{i}),m}^{⊤}. \end{array}$$
  The corresponding irreducible spherical tensor operators ${\hat{T}}_{1,m}^{n}$ (for a spin- 1/2 nucleus) and ${\hat{T}}_{1(K_{i}),m}$ (for an effective spin $K_{i}$ ) are constructed according to the standard definition in Eq. (31), using the appropriate spin quantum number ( 1/2 or $K_{i}$ , respectively). This construction is performed independently for the SABRE complex and the free substrate, using their respective spin systems and relaxation times $T_{1}$ .
6. **Construct the projected Liouvillians in the zero-quantum coherence subspaces.** The total Liouvillian superoperators for the free substrate (S) and the SABRE complex (C) are constructed by combining the respective Hamiltonian and relaxation superoperators from steps 4 and 5:
56 $${\hat{\hat{L}}}_{S,C}(t)=-i{\hat{\hat{H}}}_{S,C}(t)+{\hat{\hat{\Gamma }}}_{S,C},$$
  where ${\hat{\hat{H}}}_{S,C}(t)$ is defined by Eq. (53) and ${\hat{\hat{\Gamma }}}_{S,C}$ by Eq. (54). Following Eq. (41), we then form the auxiliary superoperators ${\hat{\hat{A}}}_{S,C}(t)$ for each system. Finally, the superoperators ${\hat{\hat{A}}}_{S,C}(t)$ , see Eq. (42), are projected onto the zero-quantum coherence (ZQC) subspaces of the free substrate ( ${\text{ZQC}}_{S}$ ) and the complex ( ${\text{ZQC}}_{C}$ ), respectively:
57 $${\hat{\hat{A}}}_{S}^{\text{ZQC}}(t)=\left({\hat{s}}_{i}\left|{\hat{\hat{A}}}_{S}(t)\right|{\hat{s}}_{j}\right),{\hat{s}}_{i},{\hat{s}}_{j}\in {\text{ZQC}}_{S},$$
  where the brackets denote the scalar product in Liouville space, ${\hat{s}}_{i,j}$ values are the basis operators in the ${\text{ZQC}}_{S}$ subspace (see Eq. 26), and indices i,j run over all possible values in the ${\text{ZQC}}_{S}$ subspace. In the same manner, we introduce
58 $${\hat{\hat{A}}}_{C}^{\text{ZQC}}(t)=\left({\hat{c}}_{i}\left|{\hat{\hat{A}}}_{C}(t)\right|{\hat{c}}_{j}\right),{\hat{c}}_{i},{\hat{c}}_{j}\in {\text{ZQC}}_{C},$$
  with ${\hat{c}}_{i,j}$ being the basis operators in the ${\text{ZQC}}_{C}$ subspace.
7. **Construct the chemical exchange superoperators.** The chemical exchange superoperators ${\hat{\hat{S}}}_{{\text{TrH}}_{2}}^{\text{ZQC}}$ and ${\hat{\hat{S}}}_{\text{Kron}}^{\text{ZQC}}$ , which describe the reversible binding dynamics between the free substrate and the complex, are constructed following the formalism detailed in the supporting information of Knecht et al. (2016). These exchange superoperators are then projected to act between the previously defined zero-quantum coherence subspaces of the free substrate ( ${\text{ZQC}}_{S}$ ) and the complex ( ${\text{ZQC}}_{C}$ ). This yields the following final projected exchange superoperators:
59 $$\begin{array}{rl} & {\hat{\hat{S}}}_{{\text{TrH}}_{2}}^{\text{ZQC}}=\left({\hat{s}}_{i}\left|{\hat{\hat{S}}}_{{\text{TrH}}_{2}}\right|{\hat{c}}_{j}\right),\\ & {\hat{\hat{S}}}_{\text{Kron}}^{\text{ZQC}}=\left({\hat{c}}_{j}\left|{\hat{\hat{S}}}_{\text{Kron}}\right|{\hat{s}}_{i}\right),\\ & \text{with }{\hat{s}}_{i}\in {\text{ZQC}}_{S},{\hat{c}}_{j}\in {\text{ZQC}}_{C}, \end{array}$$
  where the basis operators of the ZQC subspaces, ${\hat{s}}_{i},{\hat{c}}_{j}$ , are introduced in Eqs. (57) and (58). These superoperators correctly couple the reduced-dimensionality state representations of the two chemical species.
8. **Build and solve the SABRE master equation in the ZQC subspaces.** The time-dependent master equation, Eq. (47), is then solved by numerical integration, using the constructed ZQC SABRE matrix and appropriate initial conditions; see Eq. (43). The solution yields the time-evolving density matrices of the free substrate and the complex, confined to their respective ZQC subspaces: ${\hat{\rho }}_{S}(t)\in {\text{ZQC}}_{S}$ , ${\hat{\rho }}_{C}(t)\in {\text{ZQC}}_{C}$ . A key feature of this representation is that all matrix elements of the density matrices outside these subspaces are identically zero.
9. **Calculate the physical observables.** The primary quantities of interest are the expectation values of spin operators for the free substrate. These are calculated using the reduced density matrix ${\hat{\rho }}_{S}(t)$ obtained in step 8. More precisely, for a given set of effective high-spin quantum numbers $\left(K_{1},K_{2},\ldots \right)$ , we operate with the corresponding density matrix ${\hat{\rho }}_{\left(S,K_{1},K_{2},\ldots \right)}(t)$ . The expectation value of an observable operator ${\hat{O}}_{\left(K_{1},K_{2},\ldots \right)}$ (e.g., magnetization along a specific axis) is computed as
60 $$\langle {\hat{O}}_{K_{1},K_{2},\ldots }\rangle (t)=\frac{\text{Tr}\{{\hat{O}}_{K_{1},K_{2},\ldots }\cdot {\hat{\rho }}_{S,K_{1},K_{2},\ldots }(t)\}}{\text{Tr}\left\{{\hat{\rho }}_{S,K_{1},K_{2},\ldots }(t)\right\}},$$
  where the trace is taken over the Hilbert space of the free substrate's spin system. The effective-spin description represents a statistical mixture of different total spin states for each group of magnetically equivalent nuclei. Therefore, the final, physically observable $\hat{O}$ expectation value is obtained by summing the results from Eq. (60) over all possible combinations $\left(K_{1},K_{2},\ldots \right)$ , weighted by their respective statistical weights:
61 $$\langle \hat{O}\rangle (t)=\sum_{K_{1},K_{2},\ldots }g_{K_{1},K_{2},\ldots }\;\langle {\hat{O}}_{K_{1},K_{2},\ldots }\rangle (t),$$
  where the total statistical weights are defined as $g_{K_{1},K_{2},\ldots }=g_{K_{1}}\cdot g_{K_{2}}\cdots$ . The individual factors $g_{K_{i}}$ for a group of $P_{i}$ magnetically equivalent spin- 1/2 nuclei are defined by Eqs. (3) and (7).

### 3.2 Computational resources

All numerical simulations and matrix manipulations were performed using the in-house Python code. The calculations were executed on a standard desktop workstation with the following specifications: CPU 2 × Intel Xeon E5-2620 v3 at 2.40 GHz (12 cores, 24 threads) and RAM 256 GB DDR4.

The most memory-intensive step of our calculations was the storage of the ZQC SABRE matrix in the compressed sparse row (CSR) format for a system of N=14 nuclear spins, represented by a matrix of dimension $∼3.3\times 10^{6}\times 3.3\times 10^{6}$ . This required approximately 7 GB of RAM. The total computation time per magnetic field with this spin system was about 3.5 h. The total computation time of the ZULF SABRE spectrum was about 100 h.

All scalar J -coupling constants and longitudinal $T_{1}$ -relaxation times used in the simulations are provided in Appendix E. In all cases, we considered the complex to be a single substrate molecule and two hydride ligands.

## 4 Results and discussion

This section validates the ZQC subspace reduction method for computationally tractable, small spin systems by demonstrating that it yields results identical to those of the full, exact calculation while providing a dramatic speedup ( ∼30 –50 fold). This rigorous benchmark establishes the accuracy of the reduction. Furthermore, for a system of fully non-equivalent spins (representing the worst-case scenario for the reduction), the dimension of the Liouville space matrices is reduced by a factor of $O(\sqrt{N})$ , and the computational time is reduced by a factor of O(N) compared to full Liouville space simulations, where N is the number of substrate spins, underscoring the scalability of the proposed approach.

**Figure 5:**
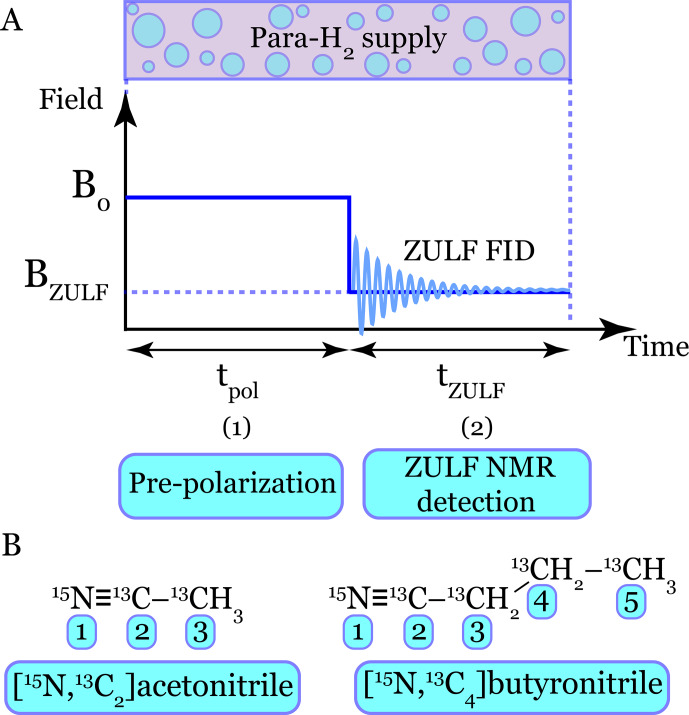
Schematic of the ZULF SABRE experiment and molecular systems. **(A)** The two-stage zero-to-ultralow field (ZULF) SABRE protocol: (1) polarization generation at a magnetic field $B_{0}∼1$ µT for a duration $t_{\text{pol}}$ and (2) ZULF NMR signal detection during the interval $t_{\text{ZULF}}$ at a residual near-zero field $B_{\text{ZULF}}∼0.01$ µT . The ZULF NMR signal is obtained via Fourier transform of ZULF free induction decay (ZULF FID). Parahydrogen bubbling is maintained continuously throughout both stages. **(B)** Chemical structures of the isotopically labeled substrates used in the simulations: [ ${}^{15}N$ , ${}^{13}C_{2}$ ]acetonitrile (6 spins) and [ ${}^{15}N$ , ${}^{13}C_{4}$ ]butyronitrile (12 spins). The numbers in parentheses indicate the total number of spin- 1/2 nuclei in each system.

For the large spin systems, where full simulations are computationally prohibitive, the validated ZQC method becomes an essential tool. Its performance gain unlocks the modeling of multi-spin systems, as illustrated in Fig. 5: panel A shows the zero-to-ultralow field (ZULF) SABRE experimental protocol, while panel B presents the two studied substrates: the small system ([ ${}^{15}N$ , ${}^{13}C_{2}$ ]acetonitrile, 6 spins) and the large system ([ ${}^{15}N$ , ${}^{13}C_{4}$ ]butyronitrile, 12 spins).

Using this approach, we address two central aspects of ZULF SABRE (see Fig. 5A): (1) calculating the magnetic field dependence of hyperpolarization to identify the optimal polarization field $B_{0}$ and (2) simulating the resulting ZULF NMR spectra for direct comparison with the experiment.

### 4.1 Validation of the ZQC reduction method: [ ${}^{15}N$ , ${}^{13}C_{2}$ ]acetonitrile

The [ ${}^{15}N$ , ${}^{13}C_{2}$ ]acetonitrile molecule constitutes a relatively small spin system (6 spins in the substrate, 8 spins in the SABRE complex), allowing for a direct numerical benchmark between the exact, unreduced calculation and the ZQC reduction approach.

**Figure 6:**
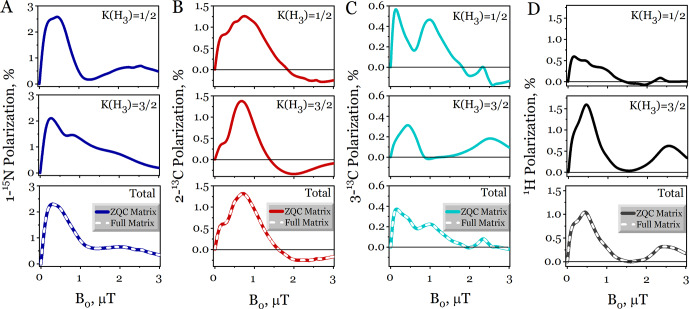
Magnetic field dependence of the hyperpolarization in free [ ${}^{15}N$ , ${}^{13}C_{2}$ ]acetonitrile computed via the ZQC reduction method. The panels correspond to **(A)** 1- ${}^{15}N$ , **(B)** 2- ${}^{13}C$ , **(C)** 3- ${}^{13}C$ , and **(D)** ${}^{1}H$ nuclei. For each nucleus, results are shown for three representations of the methyl ( ${\mathrm{CH}}_{3}$ ) group: top row – fixed effective spin K=1/2 for the three equivalent protons, middle row – fixed effective spin K=3/2 , and bottom row – weighted statistical average over K=1/2 and K=3/2 states with weights $g_{1/2}=g_{3/2}=1/2$ . In the bottom-row panel, the dashed white lines show the result of the exact, full Liouville space calculation (without any reduction) for the complete spin system, while the solid lines correspond to the ZQC-reduced calculation. The simulation parameters are the dissociation rate constant $k_{d}=10$ $s^{-1}$ , catalyst to substrate ratio [C]/[S]=0.1 , and polarization time $t_{\text{pol}}=1$ s.

First, we simulated the first stage of the ZULF SABRE experiment (Fig. 5A), the magnetic field dependence of the polarization in [ ${}^{15}N$ , ${}^{13}C_{2}$ ]acetonitrile, by varying the polarization field $B_{0}$ over a range of microtesla values. We specifically calculated the net polarization of each nucleus, corresponding to the observable operator ${\hat{I}}_{z}$ . The resulting magnetic field dependences for the different nuclei of acetonitrile are presented in Fig. 6: 1- ${}^{15}N$ (Fig. 6A), 2- ${}^{13}C$ (Fig. 6B), 3- ${}^{13}C$ (Fig. 6C), and the methyl protons (Fig. 6D).

For each nucleus, the ZQC-reduced calculation was performed for two fixed effective spin states of the three equivalent protons in the $-{\mathrm{CH}}_{3}$ group: K=1/2 (top row) and K=3/2 (middle row). The final, physically observable dependence (bottom row) was obtained by statistically averaging these two results with equal weights, $g_{1/2}=g_{3/2}=1/2$ , as prescribed by Eqs. (3), (7), and (61). For validation, the results of the exact, full SABRE matrix calculation (without any reduction) are included in the bottom row of Fig. 6 as dashed lines. They are virtually indistinguishable from the results obtained with the ZQC reduction, demonstrating a negligible relative numerical residual of the order of $10^{-5}$ .

Crucially, a direct benchmark comparison of the different computational approaches is summarized in Table 1. The ZQC reduction method achieves a 34-fold reduction in computation time compared to the full calculation. For reference, Table 1 also includes the intermediate case using only effective-spin reduction (without ZQC projection), which provides a smaller, 6-fold speedup. Notably, the observed speedup roughly scales with the ratio of the matrix dimensions, confirming that the ZQC projection effectively reduces the computational cost in proportion to the reduced size of the Liouville space block.

**Table 1:** Computation times per magnetic field point for three approaches: (1) the full SABRE matrix, (2) the SABRE matrix with effective-spin reduction of the equivalent nuclei in the methyl or methylene groups (denoted as the K-SABRE matrix), and (3) the ZQC-reduced SABRE matrix (denoted by boldface).

Matrix type	Maximum dimension^a^	Computation time
Full SABRE	69 632	54 s
K-SABRE	17 408	9 s
**ZQC SABRE**	**3034**	**1.6 s**
${[}^{15}\text{N}{,}^{13}{\text{C}}_{2}]$ acetonitrile + H_2_: 8 spins^b^		
Full SABRE	$2.9\times 10^{8}$	∼300 h
K-SABRE	$2.3\times 10^{7}$	∼24 h
**ZQC SABRE**	$3.3\times 10^{6}$	**3.5 h**
${[}^{15}\text{N}{,}^{13}{\text{C}}_{4}]$ butyronitrile + H_2_: 14 spins^c^		

^a^ The maximum dimension is the largest matrix size for the system, corresponding to the highest possible effective spins of the equivalent groups: K=3/2 ( ${\mathrm{CH}}_{3}$ ) for acetonitrile and $K_{1}=1$ , $K_{2}=1$ , and $K_{3}=3/2$ (two ${\mathrm{CH}}_{2}$ and one ${\mathrm{CH}}_{3}$ ) for butyronitrile.^b^ For the 8-spin system, the ZQC SABRE matrix reduces the computation time ∼ 34-fold.^c^ For the 14-spin system, the ZQC SABRE matrix reduces the computation time ∼ 86-fold.

**Figure 7:**
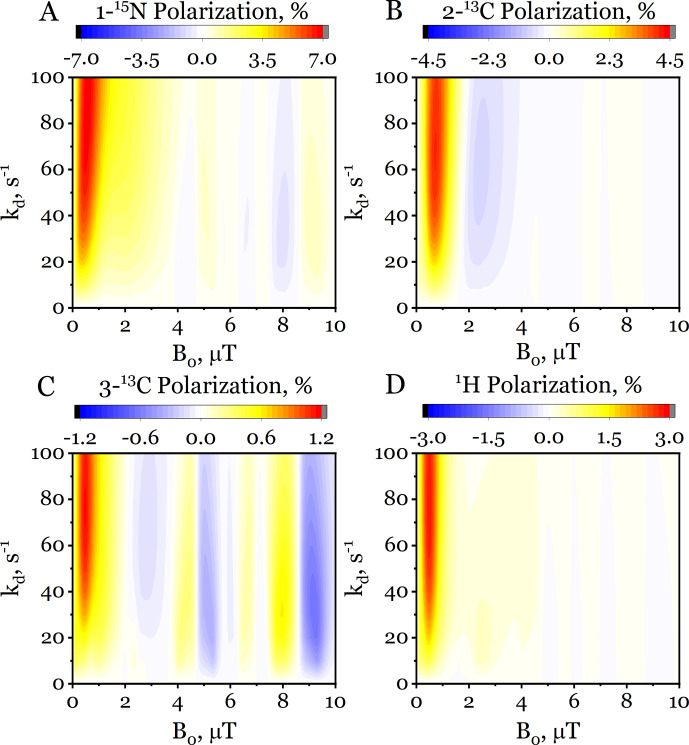
Two-dimensional dependence of the hyperpolarization in free [ ${}^{15}N$ , ${}^{13}C_{2}$ ]acetonitrile on the dissociation rate constant $k_{d}$ and the polarization transfer field $B_{0}$ , computed via the ZQC reduction method. The panels correspond to **(A)** 1- ${}^{15}N$ , **(B)** 2- ${}^{13}C$ , **(C)** 3- ${}^{13}C$ , and **(D)** ${}^{1}H$ nuclei. The simulation parameters are catalyst to substrate ratio [C]/[S]=0.1 and polarization time $t_{\text{pol}}=1$ s.

The obtained magnetic field dependences exhibit broad profiles (extending up to ∼3 µT ) and a complex structure featuring multiple extrema and sign changes. This nontrivial behavior stems from the coherent interplay of several types of nuclei ( ${}^{15}N$ , ${}^{13}C$ , ${}^{1}H$ ) within the molecule, coupled through the network of scalar interactions. Motivated by this nontrivial magnetic field dependence, we extended the simulations to a broader range (up to 10 µT ) and examined the effect of the dissociation rate constant $k_{d}$ . The resulting two-dimensional maps of hyperpolarization are shown in Fig. 7 for each nucleus: 1- ${}^{15}N$ (Fig. 7A), 2- ${}^{13}C$ (Fig. 7B), 3- ${}^{13}C$ (Fig. 7C), and the methyl protons (Fig. 7D). Critically, the ZQC reduction renders such two-dimensional scans computationally feasible, requiring only ∼1 h despite the complexity of the simulated system, which includes 8 magnetic nuclei in the SABRE complex. Thus, the maximum polarization for all nuclei occurs at approximately 0.5 µT across the entire range of dissociation rates $k_{d}$ . Beyond this common optimum, several distinct features emerge: (1) the width of the 1- ${}^{15}N$ polarization profile increases systematically with $k_{d}$ (see Fig. 7A), (2) the 2- ${}^{13}C$ nucleus exhibits two extrema – a primary positive maximum near 0.5 µT and a secondary negative extremum at higher fields (2–3 µT ) (see Fig. 7B), and (3) the 3- ${}^{13}C$ nucleus displays an unusually complex and broad profile with multiple extrema persisting up to 10 µT . This pronounced multi-peak structure is non-typical in SABRE and directly reflects the intricate network of heteronuclear couplings within the multi-spin molecule.

As the next step, we simulated the complete ZULF SABRE spectrum using the optimized two-stage protocol: (1) pre-polarization at the optimal field $B_{0}=0.5$ µT (identified above), followed by (2) ZULF FID signal detection at a near-zero field $B_{\text{ZULF}}=0.01$ µT . The detected ZULF signal is proportional to the total z magnetization of all nuclei, represented by the operator

62 $${\hat{O}}_{\text{ZULF}}=\sum_{l}\gamma^{l}{\hat{I}}_{z}^{l},$$

where the sum runs over all spins in the substrate. A detailed procedure for simulating such ZULF NMR spectra is described in Stern and Sheberstov (2023). In brief, we computed the time-dependent expectation value $\langle {\hat{O}}_{\text{ZULF}}\rangle (t)$ , see Eqs. (60) and (61), and then obtained the frequency-domain spectrum via a standard Fourier transform.

**Figure 8:**
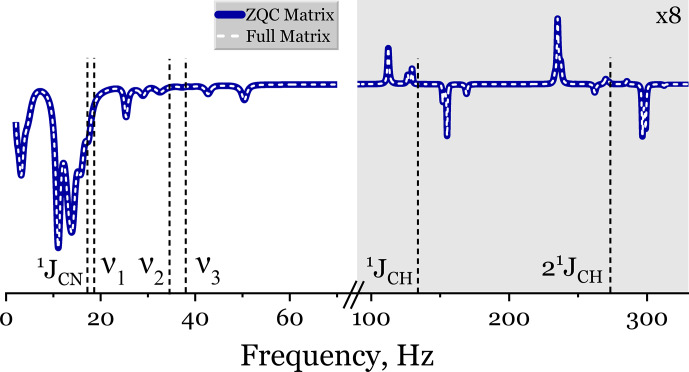
Simulated ZULF SABRE spectrum of free ${[}^{15}\text{N}{,}^{13}{\text{C}}_{2}]$ acetonitrile. The right part of the spectrum is multiplied by a factor of 8. The dashed line shows the result of the exact, full-space calculation (without ZQC or effective spin reduction) for the complete 6-spin system. The solid line corresponds to the spectrum obtained via the ZQC reduction method with effective spin treatment. The vertical dashed lines mark the characteristic spectral frequencies of acetonitrile. The high-frequency domain at ${}^{1}J_{\mathrm{CH}}$ = 136 Hz and $2\times^{1}J_{\mathrm{CH}}$ = 272 Hz and the low-frequency domain at ${}^{1}J_{\mathrm{CN}}$ , $\nu_{1}=\frac{5}{8}\left(J_{\mathrm{CC}}+3\times^{2}J_{\mathrm{CH}}\right)$ = 19.4 Hz, $\nu_{2}=\frac{3}{4}\left(J_{\mathrm{CC}}+{}^{2}J_{\mathrm{CH}}\right)$ = 36.8 Hz, and $\nu_{3}=\frac{3}{8}\left(J_{\mathrm{CC}}-5\times^{2}J_{\mathrm{CH}}\right)$ = 38.7 Hz. The simulation parameters are polarization field $B_{0}$ = 0.5 µT , detection field $B_{\mathrm{ZULF}}$ = 0.01 µT , polarization time $t_{\mathrm{pol}}$ = 10 s, acquisition time $t_{\mathrm{ZULF}}$ = 5 s, dissociation rate constant $k_{d}$ = 100 s^−1^, and catalyst to substrate ratio [C]/[S] = 0.1. The computation times were 1415 s for the full matrix and 26 s for the ZQC-reduced matrix, demonstrating a ∼ 54-fold speedup.

The resulting simulated ZULF SABRE spectrum of free [ ${}^{15}N$ , ${}^{13}C_{2}$ ]acetonitrile is shown in Fig. 8. The solid line shows the spectrum obtained with the ZQC reduction method, while the dashed line corresponds to the exact, full calculation without any reduction. The two curves are visually indistinguishable, with a negligible relative numerical difference of the order of $10^{-5}$ , providing a final validation of the accuracy of the ZQC approach. The computational speedup in this case is ∼54 -fold with the ZQC reduction.

The simulated ZULF NMR spectrum exhibits characteristic fingerprints of different scalar couplings in acetonitrile. A fully analytical description of the spectrum is nontrivial; however, qualitative insight can be obtained by considering the ${}^{13}C$ – ${}^{13}{\mathrm{CH}}_{3}$ moiety as an effective B(XA_3_) spin system, as analyzed previously (Barskiy et al., 2025). Within this simplified picture, prominent high-frequency features are expected near the couplings ${}^{1}J_{\mathrm{CH}}=136$ Hz and $2\times^{1}J_{\mathrm{CH}}$ = 272 Hz (see Appendix E for the J couplings). In addition, several low-frequency transitions are predicted in the first-order perturbation theory at $\nu_{1}=\frac{5}{8}\left(J_{\mathrm{CC}}+3\times^{2}J_{\mathrm{CH}}\right)$ = 19.4 Hz, $\nu_{2}=\frac{3}{4}\left(J_{\mathrm{CC}}+{}^{2}J_{\mathrm{CH}}\right)$ = 36.8 Hz, and $\nu_{3}=\frac{3}{8}\left(J_{\mathrm{CC}}-5\times^{2}J_{\mathrm{CH}}\right)$ = 38.7 Hz. The inclusion of the ${}^{15}N$ nucleus, which is coupled to ${}^{13}C$ via the scalar coupling ${}^{1}J_{\mathrm{CN}}$ = - 18 Hz, calls for a more detailed analysis beyond the reduced-spin model considered above. Although additional transitions arise upon inclusion of ${}^{15}N$ , the overall spectral structure remains in qualitative agreement with the simplified description. We note that a ZULF NMR spectrum of [ ${}^{15}N$ , ${}^{13}C_{2}$ ]acetonitrile with thermal pre-polarization at 1.8 T has been reported previously in Ledbetter et al. (2011). In contrast to the thermal case, the relative line intensities in the SABRE-enhanced ZULF spectrum differ substantially, reflecting the non-Boltzmann spin orders generated via SABRE.

### 4.2 Simulation of a large spin ensemble: [^15^N,^13^C_4_butyronitrile]

[ ${}^{15}N$ , ${}^{13}C_{4}$ ]butyronitrile provides a representative example of a multi-spin system comprising 12 magnetically active nuclei so that the corresponding SABRE complex contains a total of 14 spins. Direct simulations of such systems rapidly become computationally demanding on a standard desktop computer. As shown in Table 1, the computation time required for a single magnetic field point is approximately 300 h for the full Liouville space treatment and about 24 h when only the effective-spin reduction is applied, resulting in impractically long simulation times. The use of the ZQC reduction decreases the dimensionality of the problem by a factor of ∼88 (Table 1), reducing the computation time to approximately 3.5 h per field point and enabling systematic simulations of such multi-spin SABRE systems. Importantly, in our treatment we considered the $-{\mathrm{CH}}_{2}-$ and $-{\mathrm{CH}}_{3}$ groups of butyronitrile as the corresponding effective spins and then averaged the results with different statistical weights shown in Table 2.

**Table 2:** Effective-spin configurations of the $-{\mathrm{CH}}_{2}-$ and $-{\mathrm{CH}}_{3}$ groups of [ ${}^{15}N$ – ${}^{13}C_{4}$ ]butyronitrile and their corresponding statistical weights.

Effective-spin configuration	$g_{K_{1},K_{2},K_{3}}$
$K_{1}=1,K_{2}=1,K_{3}=\frac{3}{2}$	$\frac{36}{128}$
$K_{1}=1,K_{2}=1,K_{3}=\frac{1}{2}$	$\frac{36}{128}$
$K_{1}=1,K_{2}=0,K_{3}=\frac{3}{2}$	$\frac{12}{128}$
$K_{1}=1,K_{2}=0,K_{3}=\frac{1}{2}$	$\frac{12}{128}$
$K_{1}=0,K_{2}=1,K_{3}=\frac{3}{2}$	$\frac{12}{128}$
$K_{1}=0,K_{2}=1,K_{3}=\frac{1}{2}$	$\frac{12}{128}$
$K_{1}=0,K_{2}=0,K_{3}=\frac{3}{2}$	$\frac{4}{128}$
$K_{1}=0,K_{2}=0,K_{3}=\frac{1}{2}$	$\frac{4}{128}$

**Figure 9:**
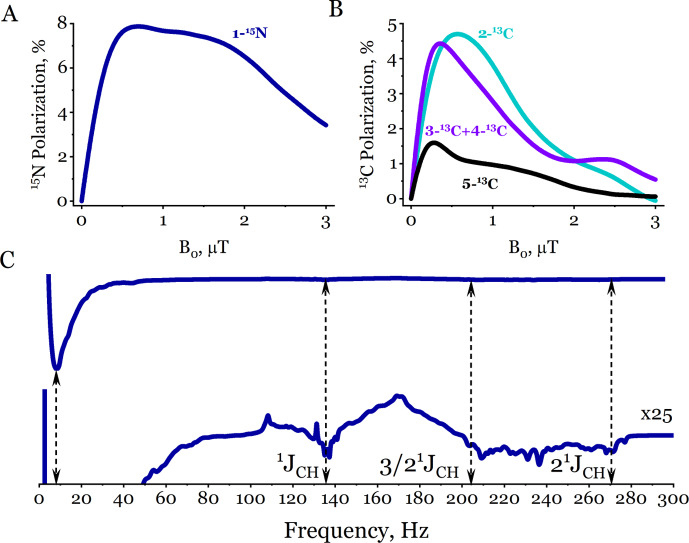
ZULF SABRE of free ${[}^{15}\text{N}{,}^{13}{\text{C}}_{4}]$ butyronitrile. **(A, B)** Simulated magnetic field dependences of the ^15^N **(A)** and ^13^C **(B)** polarization. **(C)** Simulated ZULF NMR spectrum with the ZQC reduction obtained after pre-polarization at $B_{0}$ = 0.5 µT . (Top) Full ZULF NMR spectrum, in which only the low-frequency features are visible. (Bottom) The same spectrum scaled by a factor of 25, revealing characteristic high-frequency features associated with the scalar couplings ${}^{1}J_{\mathrm{CH}}$ = 136 Hz, $3/2\times^{1}J_{\mathrm{CH}}$ = 204 Hz, and $2\times^{1}J_{\mathrm{CH}}$ = 272 Hz, indicated by dashed lines. The total computation time of the ZULF NMR spectrum was ∼ 100 h. The simulation parameters are detection field $B_{\mathrm{ZULF}}$ = 0.01 µT , polarization time $t_{\mathrm{pol}}$ = 10 s, acquisition time $t_{\mathrm{ZULF}}$ = 5 s, dissociation rate constant $k_{d}$ = 50 s^−1^, and catalyst to substrate ratio [C]/[S] = 0.37.

We first calculated the magnetic field dependences of the ${}^{15}N$ and ${}^{13}C$ polarization during the pre-polarization stage of the ZULF SABRE experiment, as shown in Fig. 9A and B, respectively. The simulated results capture the main qualitative features: (1) there is a very broad dependence of the 1- ${}^{15}N$ polarization on the pre-polarization field $B_{0}$ , extending up to ∼2 µT , reflecting the complexity of the underlying multi-spin system, and (2) the polarization levels of the different ${}^{13}C$ sites decrease along the chain. Importantly, our calculations can incorporate the actual sweeping profile of the magnetic field ramp, which is essential for capturing adiabatic effects, although doing so will increase the computational time. It should also be noted that the 3- ${}^{13}C$ and 4- ${}^{13}C$ sites are close to chemical equivalence; i.e. the difference in their chemical shifts is smaller than their J -coupling constants with other nuclei. Accordingly, we report their total magnetization (3- ${}^{13}C$ + 4- ${}^{13}C$ ) as the measured observable.

The simulated ZULF NMR spectrum is shown in Fig. 9C. The most intense signal appears in the low-frequency domain, centered around ∼10 Hz , and broadens as the number of coupled magnetic nuclei increases. In addition to this dominant low-frequency feature, characteristic fingerprints of the $-{\mathrm{CH}}_{2}-$ and $-{\mathrm{CH}}_{3}$ groups are visible: frequencies ${}^{1}J_{\mathrm{CH}}=136$ Hz and $2\times^{1}J_{\mathrm{CH}}$ = 272 Hz from the $-{\mathrm{CH}}_{3}$ group and $3/2\times^{1}J_{\mathrm{CH}}$ = 204 Hz from the $-{\mathrm{CH}}_{2}-$ group. These signals are broadened due to splitting by the J couplings present in the multi-spin system.

### 4.3 Benchmarking and scalability analysis

**Figure 10:**
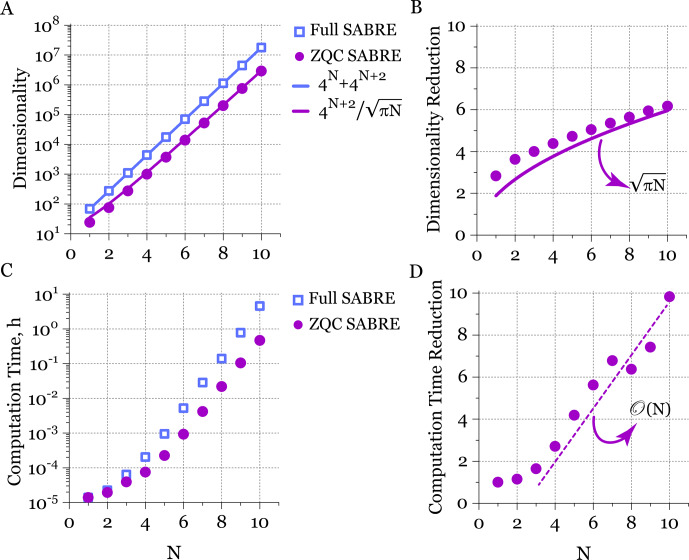
Scaling benchmark of full and ZQC-reduced Liouville space matrices for a substrate molecule with N non-equivalent spins. **(A)** Dimensionality of the full SABRE (open squares) and ZQC-reduced SABRE (filled circles) Liouville space matrices as a function of N . The solid lines represent the asymptotic scaling for $\sqrt{N}\gg 1$ . Note that the SABRE complex contains N+2 spins. **(B)** Dimensionality reduction achieved with the ZQC approach. The predicted asymptotic scaling is $\sqrt{\pi N}$ . **(C)** Computation time per magnetic field $B_{0}$ for the full SABRE (squares) and ZQC-reduced SABRE (circles) Liouville space matrices as a function of N . **(D)** Computational speedup achieved with the ZQC approach, which scales as O(N) .

In this section, we analyze the scalability of the proposed ZQC reduction approach in comparison to full Liouville space simulations as a function of the number of spins in the substrate molecule. To demonstrate its efficiency, we consider the worst-case scenario in which the substrate contains only N non-equivalent nuclei (the presence of magnetically equivalent nuclei would only further exponentially improve the scalability; see Eq. 50). The scaling benchmark as a function of N is shown in Fig. 10.

We first examine the dimensionality of the matrices, shown in Fig. 10A. For full Liouville space simulations, the dimensionality is given exactly by $\text{dim(Full SABRE)}=4^{N}+4^{N+2}$ (the SABRE complex contains N+2 spins). For the ZQC-reduced matrices, a compact asymptotic expression valid for $\sqrt{N}\gg 1$ is given by Eq. (50): $\text{dim(ZQC SABRE)}∼4^{N+2}/\sqrt{\pi N}$ . As shown in Fig. 10A, this asymptotic approximation (solid line) almost perfectly matches the dimensionality of the ZQC-reduced SABRE matrix. Although the dimensionality still grows exponentially with N , it is reduced by a factor of $\sqrt{\pi N}$ compared to the full Liouville space (see Fig. 10B).

Similarly, we investigated the dependence of the computation time per single magnetic field $B_{0}$ on the number of spins N , as shown in Fig. 10C. For small N=1,2 , the computation time of the ZQC approach is nearly identical for both matrices. For larger N , however, it scales linearly, i.e., as O(N) (Fig. 10D). This demonstrates that the reduction in computation time scales quadratically with the reduction in matrix dimension.

## 5 Conclusions

In this work, we have shown that the Hamiltonian, relaxation, and chemical exchange superoperators in SABRE systems under zero-to-ultralow-field conditions all possess well-defined symmetry with respect to the z projection of the total spin, where the z axis is defined by the external ultralow magnetic field. This approach is valid across different coupling regimes encountered in ZULF NMR, from the J -coupling-dominated to the Zeeman-dominated limits, making the approach general and broadly applicable. Importantly, this symmetry ensures that chemical exchange conserves the coherence order, allowing the dynamics to be rigorously restricted to the zero-quantum coherence (ZQC) subspace. As only operators within this subspace contribute to polarization transfer in SABRE, focusing on ZQC drastically reduces the effective dimensionality of the Liouville space while retaining a fully rigorous description of the spin dynamics.

As a first validation, we applied this approach to [ ${}^{15}N$ , ${}^{13}C_{2}$ ]acetonitrile (8 spins in the SABRE complex), reproducing full Liouville space results with excellent agreement while accelerating computations by a factor of ∼30 for magnetic field dependence calculations and ∼54 for ZULF NMR spectra. To demonstrate the scalability of the method, we further applied it to [ ${}^{15}N$ , ${}^{13}C_{4}$ ]butyronitrile (14 spins in the SABRE complex), a system that would be practically impossible to simulate with full Liouville space calculations within a reasonable time frame. Using the ZQC reduction, these simulations became feasible, providing insight into both field dependence of polarization and ZULF NMR spectra. These results confirm the benchmarking analysis for a system of N non-equivalent substrate spins: the matrix dimension is reduced roughly by a factor of $\sqrt{\pi N}$ , while the computation time is accelerated roughly by a factor of N .

Overall, the present framework provides a predictive and scalable tool for analyzing complex SABRE spin dynamics in the absence of transverse oscillating fields, which is particularly valuable for capturing the rich structure of field-dependent polarization curves and the intricate ZULF NMR spectra of multi-spin molecules. This approach therefore offers a practical pathway for designing experiments and optimizing hyperpolarization strategies in large (up to 15 spins) and chemically diverse spin systems.

## Data Availability

All data presented in this study were generated by the accompanying code, as described in the main text. No external or observational datasets were used. The generated datasets can be fully reproduced using the code provided in the “Code availability” section.

## Appendix A The coherence orders

The operator ${\hat{Q}}_{p}$ is said to have a coherence order p if it satisfies the following relation with the operator of the z projection of total spin ${\hat{F}}_{z}$ :

A1
$$[{\hat{F}}_{z},{\hat{Q}}_{p}]=p{\hat{Q}}_{p}.$$

In terms of Liouville space, where the operator ${\hat{F}}_{z}$ is mapped to the superoperator ${\hat{\hat{F}}}_{z}$ , Eq. (A1) transforms to the eigenvalue problem:

A2
$$[{\hat{F}}_{z},{\hat{Q}}_{p}]={\hat{\hat{F}}}_{z}{\hat{Q}}_{p}=p{\hat{Q}}_{p}.$$

Thus, ${\hat{Q}}_{p}$ is an eigenoperator of ${\hat{\hat{F}}}_{z}$ with eigenvalue p .

As a demonstrative example, we consider two spin- 1/2 particles and define ${\hat{F}}_{z}={\hat{I}}_{1z}+{\hat{I}}_{2z}$ . As an example, we determine the coherence order of the operator $\hat{Q}=|\alpha \alpha \rangle \langle \beta \beta |$ , where |α〉 and |β〉 denote the standard Zeeman “spin-up” and “spin-down” states, respectively. In this case, we substitute $\hat{Q}$ into Eq. (A2):

A3
$$\begin{array}{rl} {\hat{\hat{F}}}_{z}|\alpha \alpha \rangle \langle \beta \beta | & =[{\hat{F}}_{z},|\alpha \alpha \rangle \langle \beta \beta |]=[({\hat{I}}_{1z}+{\hat{I}}_{2z}),|\alpha \alpha \rangle \langle \beta \beta |]\\ & =({\hat{I}}_{1z}+{\hat{I}}_{2z})|\alpha \alpha \rangle \langle \beta \beta |-|\alpha \alpha \rangle \langle \beta \beta |({\hat{I}}_{1z}+{\hat{I}}_{2z})\\ & =(\frac{1}{2}+\frac{1}{2})|\alpha \alpha \rangle \langle \beta \beta |-|\alpha \alpha \rangle \langle \beta \beta |(-\frac{1}{2}-\frac{1}{2})\\ & =+2|\alpha \alpha \rangle \langle \beta \beta |. \end{array}$$

Therefore, |αα〉〈ββ| is a +Double-Quantum Coherence ( +DQC ) operator. In the same manner, one can find that the populations are the zero-quantum coherence (ZQC) operators:

A4
$$\begin{array}{rl} Q_{0}^{\left(1\right)} & =|\alpha \alpha \rangle \langle \alpha \alpha |,\;\;Q_{0}^{\left(2\right)}=|\alpha \beta \rangle \langle \alpha \beta |,\\ Q_{0}^{\left(3\right)} & =|\beta \alpha \rangle \langle \beta \alpha |,\;\;Q_{0}^{\left(4\right)}=|\beta \beta \rangle \langle \beta \beta |. \end{array}$$

Additionally, two more ZQC operators can be found:

A5
$$Q_{0}^{\left(5\right)}=|\alpha \beta \rangle \langle \beta \alpha |,\;\;Q_{0}^{\left(6\right)}=|\beta \alpha \rangle \langle \alpha \beta |.$$

Generally, for an arbitrary spin system, a ZQC operator is constructed as

A6
$${\hat{Q}}_{0}=|F_{z},\xi \rangle \langle F_{z},\lambda |,$$

where $F_{z}$ , which runs over all possible values for the considered spin system, and ξ,λ denote different spin states with identical values of $F_{z}$ . This construction ensures that ${\hat{\hat{F}}}_{z}{\hat{Q}}_{0}=0$ .

For example, the ZQC operators in Eqs. (A4) and (A5) can be expressed using the notations introduced in Eq. (A6):

A7
$$\begin{array}{rl} & Q_{0}^{\left(1\right)}=|1(1/2,1/2)\rangle \langle 1(1/2,1/2)|,\\ & Q_{0}^{\left(2\right)}=|0(1/2,-1/2)\rangle \langle 0(1/2,-1/2)|,\\ & Q_{0}^{\left(3\right)}=|0(-1/2,1/2)\rangle \langle 0(-1/2,1/2)|,\\ & Q_{0}^{\left(4\right)}=|-1(-1/2,-1/2)\rangle \langle -1(-1/2,-1/2)|,\\ & Q_{0}^{\left(5\right)}=|0(1/2,-1/2)\rangle \langle 0(-1/2,1/2)|,\\ & Q_{0}^{\left(6\right)}=|0(-1/2,1/2)\rangle \langle 0(1/2,-1/2)|. \end{array}$$

Here, the individual magnetic quantum numbers $m_{z}$ of two 1/2 particles are shown in brackets.

It is therefore straightforward to calculate the full number of independent ZQC operators. The total number $\text{dim}(H_{F_{z}})$ of different spin states with a fixed $F_{z}$ is given in Eq. (15). The number of ZQC operators for a fixed $F_{z}$ is given by ${\left\{\text{dim}(H_{F_{z}})\right\}}^{2}$ , as follows directly from the “ket-bra” construction of Eq. (A6). Finally, the total number of ZQC operators is obtained by summing over all possible $F_{z}$ , yielding Eq. (28).

In a broader context, the role of the coherence orders in modern NMR techniques is discussed in Ivanov et al. (2023).

## Appendix B Theorem 1. Conservation of zero coherence order in SABRE chemical reaction

### B1 Dissociation from the complex

B1
$$\text{If }{\hat{\hat{F}}}_{Cz}\;{\hat{\rho }}_{C}(t)=0\text{, then }{\hat{\hat{F}}}_{Sz}\;{\text{Tr}}_{H_{2}}\{{\hat{\rho }}_{C}(t)\}=0$$

**Proof**

The superoperator ${\hat{\hat{F}}}_{z}$ acts on the density matrix according to the following rule:

B2
$${\hat{\hat{F}}}_{z}\;\hat{\rho }(t)=[{\hat{F}}_{z},\hat{\rho }(t)].$$

Consider the following equation:

B3
$$\begin{array}{rl} {\text{Tr}}_{H_{2}} & \left\{{\hat{\hat{F}}}_{Cz}\;{\hat{\rho }}_{C}(t)\right\}\\ & ={\text{Tr}}_{H_{2}}\left\{\left({\hat{\hat{F}}}_{Sz}+{\hat{\hat{F}}}_{H_{2}z}\right)\;{\hat{\rho }}_{C}(t)\right\}\\ & ={\text{Tr}}_{H_{2}}\left\{{\hat{\hat{F}}}_{Sz}\;{\hat{\rho }}_{C}(t)\right\}+{\text{Tr}}_{H_{2}}\left\{{\hat{\hat{F}}}_{H_{2}z}\;{\hat{\rho }}_{C}(t)\right\}, \end{array}$$

where we accounted for ${\hat{\hat{F}}}_{Cz}={\hat{\hat{F}}}_{Sz}+{\hat{\hat{F}}}_{H_{2}z}$ . Here, the first superoperator acts only on the substrate’s spins, whereas the second superoperator acts only on the spins of the hydrides.

At first, we prove the following relation:

B4
$${\text{Tr}}_{H_{2}}\left\{{\hat{\hat{F}}}_{H_{2}z}\;{\hat{\rho }}_{C}(t)\right\}=0.$$

We first use the definition given in Eq. (B1):

B5
$$\begin{array}{rl} {\text{Tr}}_{H_{2}} & \left\{{\hat{\hat{F}}}_{H_{2}z}\;{\hat{\rho }}_{C}(t)\right\}\\ & ={\text{Tr}}_{H_{2}}\{[{\hat{F}}_{H_{2}z},{\hat{\rho }}_{C}(t)]\}\\ & ={\text{Tr}}_{H_{2}}\{{\hat{F}}_{H_{2}z}\;{\hat{\rho }}_{C}(t)\}-{\text{Tr}}_{H_{2}}\{{\hat{\rho }}_{C}(t)\;{\hat{F}}_{H_{2}z}\}. \end{array}$$

The operator ${\hat{F}}_{H_{2}z}$ is separable in the basis set of the complex since it acts only on the states of the hydrides:

B6
$$\begin{array}{rl} {\hat{F}}_{H_{2}z} & =\sum_{i,j}\{{\hat{F}}_{H_{2}z}{\}}_{i,j}\;|i\rangle \langle j|⊗\hat{1}\\ & =\sum_{i,j}\{{\hat{F}}_{H_{2}z}{\}}_{i,j}\;|i\rangle \langle j|⊗\sum_{\xi }|\xi \rangle \langle \xi |\\ & =\sum_{i,j,\xi }\{{\hat{F}}_{H_{2}z}{\}}_{i,j}\;|i\rangle |\xi \rangle \langle j|\langle \xi |, \end{array}$$

where the Latin letters denote the states of the hydrides, and the Greek letters denote the state of the substrate in the complex. Now we consider the density matrix of the complex in this product basis set:

B7
$${\hat{\rho }}_{C}(t)=\sum_{i,j,\xi ,\mu }\{{\hat{\rho }}_{C}(t){\}}_{i\xi ,j\mu }\;|i\rangle |\xi \rangle \langle j|\langle \mu |.$$

Now we consider the matrix elements of ${\hat{F}}_{H_{2}z}{\hat{\rho }}_{C}$ using Eqs. (B6) and (B7):

B8
$$\{{\hat{F}}_{H_{2}z}{\hat{\rho }}_{C}{\}}_{k\alpha ,l\beta }=\sum_{j}\{{\hat{F}}_{H_{2}z}{\}}_{k,j}\{{\hat{\rho }}_{C}(t){\}}_{j\alpha ,l\beta }.$$

We then take the partial trace over the states of the hydrides in Eq. (B8):

B9
$$\begin{array}{rl} {\left\{{\text{Tr}}_{H_{2}}\{{\hat{F}}_{H_{2}z}{\hat{\rho }}_{C}(t)\}\right\}}_{\alpha ,\beta } & =\sum_{k}\{{\hat{F}}_{H_{2}z}{\hat{\rho }}_{C}(t){\}}_{k\alpha ,k\beta }\\ & =\sum_{k,j}\{{\hat{F}}_{H_{2}z}{\}}_{k,j}\{{\hat{\rho }}_{C}(t){\}}_{j\alpha ,k\beta }. \end{array}$$

The matrix elements of ${\hat{\rho }}_{C}(t){\hat{F}}_{H_{2}z}$ are defined as follows:

B10
$$\{{\hat{\rho }}_{C}(t){\hat{F}}_{H_{2}z}{\}}_{k\alpha ,l\beta }=\sum_{j}\{{\hat{\rho }}_{C}(t){\}}_{k\alpha ,j\beta }\{{\hat{F}}_{H_{2}z}{\}}_{j,l}.$$

The partial trace of Eq. (B10) leads to the following result:

B11
$$\begin{array}{rl} \{{\text{Tr}}_{H_{2}} & \{{\hat{\rho }}_{C}(t){\hat{F}}_{H_{2}z}\}{\}}_{\alpha ,\beta }\\ & =\sum_{k}\{{\hat{\rho }}_{C}(t){\hat{F}}_{H_{2}z}{\}}_{k\alpha ,k\beta }\\ & =\sum_{k,j}\{{\hat{\rho }}_{C}(t){\}}_{k\alpha ,j\beta }\{{\hat{F}}_{H_{2}z}{\}}_{j,k}\\ & {=}_{\left(j↔k\right)}\sum_{k,j}\{{\hat{F}}_{H_{2}z}{\}}_{k,j}\{{\hat{\rho }}_{C}(t){\}}_{j\alpha ,k\beta }. \end{array}$$

Now we subtract Eq. (B11) from Eq. (B9), which gives Eq. (B4):

B12
$$\begin{array}{rl} {\text{Tr}}_{H_{2}} & \left\{{\hat{\hat{F}}}_{H_{2}z}\;{\hat{\rho }}_{C}(t)\right\}\\ & ={\text{Tr}}_{H_{2}}\{[{\hat{F}}_{H_{2}z},{\hat{\rho }}_{C}(t)]\}\\ & ={\text{Tr}}_{H_{2}}\{{\hat{F}}_{H_{2}z}{\hat{\rho }}_{C}(t)\}-{\text{Tr}}_{H_{2}}\{{\hat{\rho }}_{C}(t){\hat{F}}_{H_{2}z}\}\equiv 0. \end{array}$$

We then apply Eq. (B4) to Eq. (B3):

B13
$${\text{Tr}}_{H_{2}}\left\{{\hat{\hat{F}}}_{Cz}\;{\hat{\rho }}_{C}(t)\right\}={\text{Tr}}_{H_{2}}\left\{{\hat{\hat{F}}}_{Sz}\;{\hat{\rho }}_{C}(t)\right\}.$$

From the statement of the theorem, we have ${\hat{\hat{F}}}_{Cz}\;{\hat{\rho }}_{C}(t)=0$ . Therefore, using Eq. (B13), we have

B14
$$0={\text{Tr}}_{H_{2}}\left\{{\hat{\hat{F}}}_{Sz}\;{\hat{\rho }}_{C}(t)\right\}={\hat{\hat{F}}}_{Sz}\;{\text{Tr}}_{H_{2}}\{{\hat{\rho }}_{C}(t)\},$$

where at the last stage we used the fact that ${\hat{\hat{F}}}_{Sz}$ and ${\text{Tr}}_{H_{2}}$ commute as they act on different variables. Finally, Eq. (B14) proves that zero coherence order is conserved after dissociation from the complex.

### B2 Association with the complex

B15
$$\text{If }{\hat{\hat{F}}}_{Sz}\;{\hat{\rho }}_{S}(t)=0\text{, then }{\hat{\hat{F}}}_{Cz}\{{\hat{\rho }}_{S}(t)⊗{\hat{\rho }}_{{\text{pH}}_{2}}\}=0$$

**Proof**

This is trivial since for parahydrogen (the singlet nuclear spin state with zero coherence order), we have ${\hat{\hat{F}}}_{H_{2}z}\;{\hat{\rho }}_{{\text{pH}}_{2}}=0$ . Therefore,

B16
$$\begin{array}{rl} {\hat{\hat{F}}}_{Cz} & \;\{{\hat{\rho }}_{S}(t)⊗{\hat{\rho }}_{{\text{pH}}_{2}}\}\\ & =\left({\hat{\hat{F}}}_{Sz}+{\hat{\hat{F}}}_{H_{2}z}\right)\;\{{\hat{\rho }}_{S}(t)⊗{\hat{\rho }}_{{\text{pH}}_{2}}\}\\ & =\underset{0}{\underset{︸}{{\hat{\hat{F}}}_{Sz}\;{\hat{\rho }}_{S}(t)}}⊗{\hat{\rho }}_{{\text{pH}}_{2}}+{\hat{\rho }}_{S}(t)⊗\underset{0}{\underset{︸}{{\hat{\hat{F}}}_{H_{2}z}\;{\hat{\rho }}_{{\text{pH}}_{2}}}}=0. \end{array}$$

## Appendix C Theorem 2. Restriction of SABRE dynamics to the zero-quantum coherence subspaces

If the following conditions are fulfilled for the Liouvillians and the initial state of the SABRE system,

C1F^^Cz,L^^C(t)=0 for any t,

C2F^^Sz,L^^S(t)=0 for any t,

C3F^^Czρ^C(0)=0,

C4F^^Szρ^S(0)=0,

then

C5
$${\hat{\hat{F}}}_{Cz}\;{\hat{\rho }}_{C}(t)=0,{\hat{\hat{F}}}_{Sz}\;{\hat{\rho }}_{S}(t)=0\text{ for any }t.$$

**Proof**

The proof uses a numerical solution of Eq. (40) discretized on a time grid with a step dt :

C6ρ^C(dt)-ρ^C(0)=A^^C(0)ρ^C(0)dt+Wa{ρ^S(0)⊗ρ^pH2},

C7ρ^S(dt)-ρ^S(0)=A^^S(0)ρ^S(0)dt+kdTrH2{ρ^C(0)}.

We now apply ${\hat{\hat{F}}}_{Sz}$ for both sides of Eq. (C7):

C8
$$\begin{array}{rl} {\hat{\hat{F}}}_{Sz} & \;{\hat{\rho }}_{S}(dt)\\ & =\underset{0}{\underset{︸}{{\hat{\hat{F}}}_{Sz}\;{\hat{\rho }}_{S}(0)}}+\underset{0}{\underset{︸}{{\hat{\hat{F}}}_{Sz}\;{\hat{\hat{A}}}_{S}(0)\;{\hat{\rho }}_{S}(0)}}dt\\ & +k_{d}\underset{0}{\underset{︸}{{\hat{\hat{F}}}_{Sz}\;{\text{Tr}}_{H_{2}}\{{\hat{\rho }}_{C}(0)\}}}dt=0, \end{array}$$

where each term is zero:

1. ${\hat{\hat{F}}}_{Sz}\;{\hat{\rho }}_{S}(0)=[{\hat{F}}_{Sz},{\hat{\rho }}_{S}(0)]=0$ , as it follows from the initial condition of Eq. (43). According to this, ${\hat{\rho }}_{S}(0)$ is proportional to the identity matrix that commutes with any operator. Consequently, Eq. (C4) is indeed valid.
2. ${\hat{\hat{F}}}_{Sz}\;{\hat{\hat{A}}}_{S}(0)\;{\hat{\rho }}_{S}(0)={\hat{\hat{F}}}_{Sz}\;\left\{{\hat{\hat{L}}}_{S}(0)-W_{a}{\hat{\hat{1}}}_{S}\right\}{\hat{\rho }}_{S}(0)=\left\{{\hat{\hat{L}}}_{S}(0)-W_{a}{\hat{\hat{1}}}_{S}\right\}\underset{0}{\underset{︸}{{\hat{\hat{F}}}_{Sz}\;{\hat{\rho }}_{S}(0)}}=0$ Here we first used Eq. (C2) and then Eq. (C4).
3. ${\hat{\hat{F}}}_{Sz}\;{\text{Tr}}_{H_{2}}\{{\hat{\rho }}_{C}(0)\}=0$ by Eq. (B1) since for the initial state of the complex Eq. (C3), ${\hat{\hat{F}}}_{Cz}\;{\hat{\rho }}_{C}(0)=0$ .

Similarly, applying ${\hat{\hat{F}}}_{Cz}$ for both sides of Eq. (C6) and using Eqs. (C1), (C3), (C4), and (B15), we obtain

C9
$${\hat{\hat{F}}}_{Cz}\;{\hat{\rho }}_{C}(dt)=0.$$

The following proof is based on induction by dt .

**Induction hypothesis**

C10
$${\hat{\hat{F}}}_{Sz}\;{\hat{\rho }}_{S}(t_{n-1})=0,{\hat{\hat{F}}}_{Cz}\;{\hat{\rho }}_{C}(t_{n-1})=0$$

Here $t_{k}=k\cdot dt$ is the k th node of the time grid. From this, we prove that

C11
$${\hat{\hat{F}}}_{Sz}\;{\hat{\rho }}_{S}(t_{n})=0,{\hat{\hat{F}}}_{Cz}\;{\hat{\rho }}_{C}(t_{n})=0.$$

**Inductive step**

From Eqs. (40), we have

C12
$$\begin{array}{rl} {\hat{\rho }}_{C}(t_{n})-{\hat{\rho }}_{C}(t_{n-1}) & ={\hat{\hat{A}}}_{C}(t_{n-1})\;{\hat{\rho }}_{C}(t_{n-1})dt\\ & +W_{a}\;\{{\hat{\rho }}_{S}(t_{n-1})⊗{\hat{\rho }}_{{\text{pH}}_{2}}\}dt. \end{array}$$

Applying ${\hat{\hat{F}}}_{Cz}$ for both sides of Eq. (C12), we obtain the following:

C13
$$\begin{array}{rl} {\hat{\hat{F}}}_{Cz} & \;{\hat{\rho }}_{C}(t_{n})\\ & ={\hat{\hat{F}}}_{Cz}\;{\hat{\rho }}_{C}(t_{n-1})+{\hat{\hat{A}}}_{C}(t_{n-1})\;{\hat{\hat{F}}}_{Cz}\;{\hat{\rho }}_{C}(t_{n-1})dt\\ & +W_{a}\underset{0}{\underset{︸}{{\hat{\hat{F}}}_{Cz}\;\{{\hat{\rho }}_{S}(t_{n-1})⊗{\hat{\rho }}_{{\text{pH}}_{2}}\}}}dt. \end{array}$$

The last term is zero because of the induction hypothesis; see Eqs. (C10) and (B15):

C14
$${\hat{\hat{F}}}_{Cz}\;\{{\hat{\rho }}_{S}(t_{n-1})⊗{\hat{\rho }}_{{\text{pH}}_{2}}\}=0.$$

Therefore, using Eq. (C13), we prove the theorem

C15
$$\begin{array}{rl} {\hat{\hat{F}}}_{Cz} & \;{\hat{\rho }}_{C}(t_{n})\\ & =\underset{0}{\underset{︸}{{\hat{\hat{F}}}_{Cz}\;{\hat{\rho }}_{C}(t_{n-1})}}+{\hat{\hat{A}}}_{C}(t_{n-1})\underset{0}{\underset{︸}{{\hat{\hat{F}}}_{Cz}\;{\hat{\rho }}_{C}(t_{n-1})}}dt=0, \end{array}$$

since ${\hat{\hat{F}}}_{Cz}\;{\hat{\rho }}_{C}(t_{n-1})=0$ from the induction hypothesis; see Eq. (C10). Therefore, we proved that ${\hat{\hat{F}}}_{Cz}\;{\hat{\rho }}_{C}(t)=0$ for any t . Similarly, one can show that ${\hat{\hat{F}}}_{Sz}\;{\hat{\rho }}_{S}(t)=0$ for any t .

## Appendix D Asymptotics for the size of the ZQC-reduced SABRE matrix

From Eq. (28), for the dimension of a ZQC subspace we have

D1
dim(ZQC)=∑Fz=-(N-P)/2-K(N-P)/2+K{∑mk=-KKN-PFz+(N-P)/2-mk}2.

Let us rewrite the inner squared sum:

D2
dim(ZQC)=∑Fz=-(N-P)/2-K(N-P)/2+K∑mk=-KK∑mk′=-KKN-PFz+(N-P)/2-mk×N-PFz+(N-P)/2-mk′=∑mk=-KK∑mk′=-KK{∑Fz=-(N-P)/2-K(N-P)/2+KN-PFz+(N-P)/2-mk×N-PFz+(N-P)/2-mk′},

where at the last stage we changed the order of the sums. Let us consider the inner sum. We introduce new variables $t=F_{z}+(N-P)/2-m_{k}$ , $\Delta =m_{k}-m_{k}^{\prime }$ :

D3
∑Fz=-(N-P)/2-K(N-P)/2+KN-PFz+(N-P)/2-mk×N-PFz+(N-P)/2-mk′=∑t=-K-mkN-P+K-mkN-PtN-Pt+Δ.

The sum is defined only if both binomial coefficients are defined. Therefore, we require $0\leq t_{\text{binom}}\leq N-P$ and $0\leq t_{\text{binom}}+\Delta \leq N-P$ . When combined, we require $t_{\text{binom}}\in [max(0,-\Delta ),min(N-P,N-P-\Delta )]$ . For example, at Δ>0 both binomial coefficients are defined if $t_{\text{binom}}\in [0,N-P-\Delta ]$ . In turn, the sum itself is taken over $t_{\text{sum}}\in [-K-m_{k},N-P+K-m_{k}]$ . Fortunately, for all Δ we have $t_{\text{binom}}\subset t_{\text{sum}}$ , and the summation interval always covers $t_{\text{binom}}$ . In this case, Vandermonde's identity is valid:

D4
∑t=-K-mkN-P+K-mkN-PtN-Pt+Δ=2N-2PN-P+Δ=2N-2PN-P+mk-mk′.

Thus, we have

D5
dim(ZQC)=∑mk=-KK∑mk′=-KK2N-2PN-P+mk-mk′.

If $\sqrt{N-P}\gg K$ , the Gauss asymptotics is valid for the binomial coefficient

D6
2N-2PN-P+mk-mk′∼4N-Pπ(N-P)e-(mk-mk′)2/(N-P)≈4N-Pπ(N-P).

Therefore, the total sum is

D7
$$\begin{array}{rl} \text{dim}(\text{ZQC}) & \approx \frac{4^{N-P}}{\sqrt{\pi (N-P)}}\sum_{m_{k}=-K}^{K}\sum_{m_{k}^{\prime }=-K}^{K}1\\ & =\frac{(2K+1{)}^{2}}{\sqrt{\pi (N-P)}}4^{N-P}. \end{array}$$

The total dimension of the ZQC SABRE matrix is

D8
$$\begin{array}{rl} \text{dim} & \left(\text{ZQC SABRE}\right)\\ & =\text{dim}({\text{ZQC}}_{S})+\text{dim}({\text{ZQC}}_{C})\\ & ∼\frac{(2K+1{)}^{2}}{\sqrt{\pi (N-P)}}4^{N-P}+\frac{(2K+1{)}^{2}}{\sqrt{\pi (N-P+2)}}4^{N-P+2}\\ & ∼\frac{(2K+1{)}^{2}}{\sqrt{\pi (N-P)}}4^{N-P+2}. \end{array}$$

## Appendix E Simulation parameters

The J -coupling constants for the SABRE complex with acetonitrile were taken from Mewis et al. (2015); see Table E1. The J -coupling constants for [ ${}^{15}N$ , ${}^{13}C_{4}$ ]butyronitrile (Table E2) are based on our experimental measurements. These data will be discussed comprehensively in a subsequent paper (Kiryutin et al., 2026). For simplicity, in all cases we assumed that the J -coupling network between the nuclei within the substrates remains unchanged upon binding to the complex.

**Table E1:** Simulation NMR parameters used for ${[}^{15}\text{N}{,}^{13}{\text{C}}_{2}]$ acetonitrile.

J , Hz	Ha	Hb	1- ${}^{15}N$	2- ${}^{13}C$	3- ${}^{13}C$	${\mathrm{CH}}_{3}$	$T_{1}$ , s
Ha		-7.6	-25	-5	-2	-0.5	1
Hb			1	0	0	0	1
1- ${}^{15}N$				-18	1	-1.6	3 (30)
2- ${}^{13}C$					58	-9	3 (30)
3- ${}^{13}C$						136	3 (30)
${\mathrm{CH}}_{3}$							1 (1)

Ha, Hb denote the hydride ligands.The values of T_1_ in brackets correspond to the free substrate.

**Table E2:** Simulation NMR parameters used for [ ${}^{15}N$ , ${}^{13}C_{4}$ ]butyronitrile.

J , Hz	Ha	Hb	1- ${}^{15}N$	2- ${}^{13}C$	3- ${}^{13}C$	4- ${}^{13}C$	5- ${}^{13}C$	3- ${\mathrm{CH}}_{2}$	4- ${\mathrm{CH}}_{2}$	${\mathrm{CH}}_{3}$	$T_{1}$ , s
Ha		-7.6	-25.6	12	0	0	0	0	0	0	1
Hb			1.5	0	0	0	0	0	0	0	1
1- ${}^{15}N$				17.4	-2.9	0.6	0.005	1.7	0.03	-0.005	3 (30)
2- ${}^{13}C$					55.2	-2.8	3.5	-9.6	6.4	-0.03	3 (30)
3- ${}^{13}C$						33.0	-1.0	135.2	-4.1	6.4	3 (30)
4- ${}^{13}C$							34.8	-5.1	130.6	-4.5	3 (30)
5- ${}^{13}C$								4.8	-4.2	126.1	3 (30)
3- ${\mathrm{CH}}_{2}$									7.0	-0.04	1 (1)
4- ${\mathrm{CH}}_{2}$										7.4	1 (1)
${\mathrm{CH}}_{3}$											1 (1)

Ha, Hb denote the hydride ligands.The values of $T_{1}$ in brackets correspond to the free substrate.3-CH_2_/4-CH_2_ are the methylene protons at the 3-^13^C/4-^13^C positions.

## Code availability

The Python scripts used for SABRE simulations with the zero-quantum coherence reduction is available at 10.5281/zenodo.18083297 (Markelov, 2025).

## References

[bib1.bibx1] Abergel D, Palmer AG (2005). A Markov Model for Relaxation and Exchange in NMR Spectroscopy. J Phys Chem B.

[bib1.bibx2] Adams RW, Aguilar JA, Atkinson KD, Cowley MJ, Elliott PIP, Duckett SB, Green GGR, Khazal IG, López-Serrano J, Williamson DC (2009). Reversible Interactions with para-Hydrogen Enhance NMR Sensitivity by Polarization Transfer. Science.

[bib1.bibx3] Angelovski G, Tickner BJ, Wang G (2023). Opportunities and Challenges with Hyperpolarized Bioresponsive Probes for Functional Imaging using Magnetic Resonance. Nat Chem.

[bib1.bibx4] Atkinson KD, Cowley MJ, Elliott PIP, Duckett SB, Green GGR, López-Serrano J, Whitwood AC (2009). Spontaneous Transfer of Parahydrogen Derived Spin Order to Pyridine at Low Magnetic Field. J Am Chem Soc.

[bib1.bibx5] Barskiy DA, Pravdivtsev AN (2025). Magnetization and Polarization of Coupled Nuclear Spins Ensembles at High Magnetic Fields. ChemPhysChem.

[bib1.bibx6] Barskiy DA, Knecht S, Yurkovskaya AV, Ivanov KL (2019). SABRE: Chemical Kinetics and Spin Dynamics of the Formation of Hyperpolarization. Prog Nucl Mag Res Sp.

[bib1.bibx7] Barskiy DA, Tayler MCD, Marco-Rius I, Kurhanewicz J, Vigneron DB, Cikrikci S, Aydogdu A, Reh M, Pravdivtsev AN, Hövener J-B, Blanchard JW, Wu T, Budker D, Pines A (2019). Zero-Field Nuclear Magnetic Resonance of Chemically Exchanging Systems. Nat Commun.

[bib1.bibx8] Barskiy DA, Blanchard JW, Budker D, Eills J, Pustelny S, Sheberstov KF, Tayler MCD, Trabesinger AH (2025). Zero- to Ultralow-field Nuclear Magnetic Resonance. Prog Nucl Mag Res Sp.

[bib1.bibx9] Blanchard JW, Budker D (2016). Zero- to Ultralow-field NMR. eMagRes.

[bib1.bibx10] Blanchard JW, Ledbetter MP, Theis T, Butler MC, Budker D, Pines A (2013). High-Resolution Zero-Field NMR 
J
-Spectroscopy of Aromatic Compounds. J Am Chem Soc.

[bib1.bibx11] Blanchard JW, Sjolander TF, King JP, Ledbetter MP, Levine EH, Bajaj VS, Budker D, Pines A (2015). Measurement of Untruncated Nuclear Spin Interactions via Zero- to Ultralow-field Nuclear Magnetic Resonance. Phys Rev B.

[bib1.bibx12] Blanchard JW, Wu T, Eills J, Hu Y, Budker D (2020). Zero- to Ultralow-field Nuclear Magnetic Resonance 
J
-Spectroscopy with Commercial Atomic Magnetometers. J Magn Reson.

[bib1.bibx13] Boutin C, Desvaux H, Carrière M, Leteurtre F, Jamin N, Boulard Y, Berthault P (2011). Hyperpolarized 
${}^{129}\mathrm{Xe}$
 NMR Signature of Living Biological Cells. NMR Biomed.

[bib1.bibx14] Buckenmaier K, Neumann R, Bullinger F, Kempf N, Povolni P, Engelmann J, Samlow J, Hövener J-B, Scheffler K, Ortmeier A, Plaumann M, Körber R, Theis T, Pravdivtsev AN (2025). Indirect Zero-Field Nuclear Magnetic Resonance Spectroscopy. Anal Chem.

[bib1.bibx15] Buntkowsky G, Theiss F, Lins J, Miloslavina YA, Wienands L, Kiryutin A, Yurkovskaya A (2022). Recent Advances in the Application of Parahydrogen in Catalysis and Biochemistry. RSC Adv.

[bib1.bibx16] Burns MJ, Rayner PJ, Green GGR, Highton LAR, Mewis RE, Duckett SB (2015). Improving the Hyperpolarization of 
${}^{31}P$
 Nuclei by Synthetic Design. J Phys Chem B.

[bib1.bibx17] Cavallari E, Carrera C, Sorge M, Bonne G, Muchir A, Aime S, Reineri F (2018). The 
${}^{13}C$
 Hyperpolarized Pyruvate Generated by Parahydrogen Detects the Response of the Heart to Altered Metabolism in Real Time. Sci Rep.

[bib1.bibx18] Cho A, Eskandari R, Granlund KL, Keshari KR (2019). Hyperpolarized [6-
${}^{13}C$
,
${}^{15}N_{3}$
]-Arginine as a Probe for *In vivo* Arginase Activity. ACS Chem Biol.

[bib1.bibx19] Cuperlovic M, Meresi GH, Palke WE, Gerig J (2000). Spin Relaxation and Chemical Exchange in NMR Simulations. J Magn Reson.

[bib1.bibx20] Dey A, Charrier B, Martineau E, Deborde C, Gandriau E, Moing A, Jacob D, Eshchenko D, Schnell M, Melzi R, Kurzbach D, Ceillier M, Chappuis Q, Cousin SF, Kempf JG, Jannin S, Dumez J-N, Giraudeau P (2020). Hyperpolarized NMR Metabolomics at Natural 
${}^{13}C$
 Abundance. Anal Chem.

[bib1.bibx21] Doktorov AB, Ivanov KL, Lukzen NN, Morozov VA (2002). Application of the Integral Encounter Theory to the Description of Degenerate Electron Exchange Reactions. J Chem Phys.

[bib1.bibx22] Doronin S, Fel’dman E, Zenchuk A (2011). The Multiple Quantum NMR Dynamics in Systems of Equivalent Spins with a Dipolar Ordered Initial State. J Exp Theor Phys.

[bib1.bibx23] Eills J, Budker D, Cavagnero S, Chekmenev EY, Elliott SJ, Jannin S, Lesage A, Matysik J, Meersmann T, Prisner T, Reimer JA, Yang H, Koptyug IV (2023). Spin Hyperpolarization in Modern Magnetic Resonance. Chem Rev.

[bib1.bibx24] Eriksson SL, Lindale JR, Li X, Warren WS (2022). Improving SABRE Hyperpolarization with Highly Nonintuitive Pulse Sequences: Moving Beyond Avoided Crossings to Describe Dynamics. Sci Adv.

[bib1.bibx25] Feskov SV, Ivanov AI, Burshtein AI (2005). Integral Encounter Theory of Strong Electron Transfer. J Chem Phys.

[bib1.bibx26] Freeman R, Wittekoek S, Ernst R (1970). High-Resolution NMR Study of Relaxation Mechanisms in a Two-Spin System. J Chem Phys.

[bib1.bibx27] Hogben HJ, Krzystyniak M, Charnock GT, Hore PJ, Kuprov I (2011). Spinach–A Software Library for Simulation of Spin Dynamics in Large Spin Systems. J Magn Reson.

[bib1.bibx28] Hong T, Wang Y, Shao Z, Li Q, Jiang M, Peng X (2025). Femtotesla Atomic Magnetometer for Zero- and Ultralow-field Nuclear Magnetic Resonance. Magn Reson Lett.

[bib1.bibx29] Ivanov KL, Lukzen NN, Kipriyanov AA, Doktorov AB (2004). The Integral Encounter Theory of Multistage Reactions Containing Association–Dissociation Reaction Stages Part I. Kinetic Equations. Phys Chem Chem Phys.

[bib1.bibx30] Ivanov KL, Pravdivtsev AN, Yurkovskaya AV, Vieth H-M, Kaptein R (2014). The Role of Level Anti-Crossings in Nuclear Spin Hyperpolarization. Prog Nucl Mag Res Sp.

[bib1.bibx31] Ivanov KL, Madhu P, Rajalakshmi G (2023). Two-Dimensional (2D) NMR Methods.

[bib1.bibx32] Jameson G, Bruschweiler R (2021). NMR Spin Ralaxation Theory of Biomolecules Undergoing Highly Asymmetric Exchange with Large Interaction Partners. J Chem Theory Comput.

[bib1.bibx33] Jiang M, Frutos RP, Wu T, Blanchard JW, Peng XH, Budker D (2019). Magnetic Gradiometer for the Detection of Zero- to Ultralow-field Nuclear Magnetic Resonance. Phys Rev Appl.

[bib1.bibx34] Keshari KR, Wilson DM (2014). Chemistry and Biochemistry of 
${}^{13}C$
 Hyperpolarized Magnetic Resonance Using Dynamic Nuclear Polarization. Chem Soc Rev.

[bib1.bibx35] Kim Y, Chen H-Y, Nickles T, Shkliar I, Dang D, Slater J, Wang C, Gordon JW, Tan CT, Suszczynski C, Maddali S, Gaunt A, Chen R, Villanueva-Meyer J, Xu D, Larson PEZ, Kurhanewicz J, Bok RA, Chang S, Vigneron DB (2025). Translation of Hyperpolarized [
${}^{13}C$
,
${}^{15}N_{2}$
]Urea MRI for Novel Human Brain Perfusion Studies. npj Imaging.

[bib1.bibx36] Kiryutin A, Zhukov I, Markelov D, Dyke EV, Yurkovskaya A, Barskiy D (2026). High-Field NMR Characterization and Indirect 
J
-Spectroscopy of a Nuclear Spin Chain [U-
${}^{13}C$
,
${}^{15}N$
]-butyronitrile. arXiv [preprint].

[bib1.bibx37] Kiryutin AS, Yurkovskaya AV, Kaptein R, Vieth H-M, Ivanov KL (2013). Evidence for Coherent Transfer of Parahydrogen-Induced Polarization at Low Magnetic Fields. J Phys Chem Lett.

[bib1.bibx38] Kiryutin AS, Yurkovskaya AV, Zimmermann H, Vieth H-M, Ivanov KL (2018). Complete Magnetic Field Dependence of SABRE-Derived Polarization. Magn Reson Chem.

[bib1.bibx39] Kiryutin AS, Zhukov IV, Ferrage F, Bodenhausen G, Yurkovskaya AV, Ivanov KL (2021). Sequential Assignment of NMR Spectra of Peptides at Natural Isotopic Abundance with Zero- and Ultralow-field TOCSY. Phys Chem Chem Phys.

[bib1.bibx40] Kiryutin AS, Markelov DA, Matsulevich ZV, Kosenko ID, Kireev NV, Godovikov IA, Yurkovskaya AV (2025). Microtesla Signal Amplification by Reversible Exchange Enables Simultaneous over 5 % Polarization of 
${}^{77}\mathrm{Se}$
 and 
${}^{15}N$
 at Natural Abundance in a Selenium–Nitrogen Heterocycle. J Am Chem Soc.

[bib1.bibx41] Knecht S, Ivanov KL (2019). Quantitative Quantum Mechanical Approach to SABRE Hyperpolarization at High Magnetic Fields. J Chem Phys.

[bib1.bibx42] Knecht S, Pravdivtsev AN, Hovener JB, Yurkovskaya AV, Ivanov KL (2016). Quantitative Description of the SABRE Process: Rigorous Consideration of Spin Dynamics and Chemical Exchange. RSC Adv.

[bib1.bibx43] Knecht S, Barskiy DA, Buntkowsky G, Ivanov KL (2020). Theoretical Description of Hyperpolarization Formation in the SABRE-Relay Method. J Chem Phys.

[bib1.bibx44] Kowalewski J, Mäler L (2006). Nuclear Spin Relaxation in Liquids: Theory, Experiments, and Applications.

[bib1.bibx45] Kozinenko VP, Kiryutin AS, Yurkovskaya AV (2025). SLIC-SABRE at Microtesla Fields Enables High Levels of Nuclear Spin Polarization Without Magnetic Shielding. Chem Methods.

[bib1.bibx46] Kuhn LT, Weber S, Bargon J, Parella T, Pérez-Trujillo M (2023). Hyperpolarization-Enhanced NMR Spectroscopy of Unaltered Biofluids using Photo-CIDNP. Anal Chem.

[bib1.bibx47] Kuprov I (2018). Large-Scale NMR Simulations in Liquid State: A Tutorial. Magn Reson Chem.

[bib1.bibx48] Kuprov I, Wagner-Rundell N, Hore P (2007). Polynomially Scaling Spin Dynamics Simulation Algorithm Based on Adaptive State-Space Restriction. J Magn Reson.

[bib1.bibx49] Ledbetter MP, Crawford CW, Pines A, Wemmer DE, Knappe S, Kitching J, Budker D (2009). Optical Detection of NMR 
J
-Spectra at Zero Magnetic Field. J Magn Reson.

[bib1.bibx50] Ledbetter MP, Theis T, Blanchard JW, Ring H, Ganssle P, Appelt S, Blümich B, Pines A, Budker D (2011). Near-Zero-Field Nuclear Magnetic Resonance. Phys Rev Lett.

[bib1.bibx51] Levitt MH (2008). Spin Dynamics: Basics of Nuclear Magnetic Resonance.

[bib1.bibx52] Li X, Lindale JR, Eriksson SL, Warren WS (2022). SABRE Enhancement with Oscillating Pulse Sequences. Phys Chem Chem Phys.

[bib1.bibx53] Limbach H-H (1991). Dynamic NMR Spectroscopy in the Presence of Kinetic Hydrogen/Deuterium Isotope Effects. Deuterium and Shift Calculation.

[bib1.bibx54] Lindale JR, Eriksson SL, Tanner CP, Warren WS (2020). Infinite-Order Perturbative Treatment for Quantum Evolution with Exchange. Sci Adv.

[bib1.bibx55] Mamone S, Floreani F, Faramawy AM, Graiff C, Franco L, Ruzzi M, Tubaro C, Stevanato G (2025). (De)coding SABRE of [1-
${}^{13}C$
] Pyruvate. Phys Chem Chem Phys.

[bib1.bibx56] Markelov D (2025). Scripts for scalable modeling of SABRE with zero-quantum projection, Zenodo [code].

[bib1.bibx57] Markelov DA, Kozinenko VP, Knecht S, Kiryutin AS, Yurkovskaya AV, Ivanov KL (2021). Singlet to Triplet Conversion in Molecular Hydrogen and Its Role in Parahydrogen Induced Polarization. Phys Chem Chem Phys.

[bib1.bibx58] Markelov DA, Kozinenko VP, Kiryutin AS, Yurkovskaya AV (2024). High-Field SABRE Pulse Sequence Design for Chemically Non-Equivalent Spin Systems. J Chem Phys.

[bib1.bibx59] Markelov DA, Kiryutin AS, Borisov AV, Kosenko ID, Godovikov IA, Yurkovskaya AV (2025). ${}^{77}\mathrm{Se}$
 Hyperpolarization Enabled by Reversible Parahydrogen Exchange and Audio-Frequency Magnetic Fields at 0.1 mT. J Phys Chem Lett.

[bib1.bibx60] McBride SJ, Pike M, Curran E, Zavriyev A, Adebesin B, Tucker L, Harzan JM, Senanayake IM, Shen S, Abdulmojeed M, Theiss F, Boele T, Gade TP, Duckett S, Goodson BM, Rosen MS, Chekmenev EY, Yuan H, Dedesma C, Kadlecek S, Theis T, TomHon P (2025). Scalable Hyperpolarized MRI Enabled by Ace-SABRE of [1-
${}^{13}C$
]Pyruvate. Angew Chem Int Edit.

[bib1.bibx61] Messiah A (1962). Quantum Mechanics.

[bib1.bibx62] Mewis RE, Green RA, Cockett MC, Cowley MJ, Duckett SB, Green GG, John RO, Rayner PJ, Williamson DC (2015). Strategies for the Hyperpolarization of Acetonitrile and Related Ligands by SABRE. J Phys Chem B.

[bib1.bibx63] Mishra A, Pariani G, Oerther T, Schwaiger M, Westmeyer GG (2016). Hyperpolarized Multi-Metal 
${}^{13}C$
-Sensors for Magnetic Resonance Imaging. Anal Chem.

[bib1.bibx64] Olaru AM, Robertson TBR, Lewis JS, Antony A, Iali W, Mewis RE, Duckett SB (2018). Extending the Scope of 
${}^{19}F$
 Hyperpolarization through Signal Amplification by Reversible Exchange in MRI and NMR Spectroscopy. ChemistryOpen.

[bib1.bibx65] Park H, Wang Q (2022). State-of-the-art Accounts of Hyperpolarized 
${}^{15}N$
-Labeled Molecular Imaging Probes for Magnetic Resonance Spectroscopy and Imaging. Chem Sci.

[bib1.bibx66] Petersen S, Nagel L, Groß PR, de Maissin H, Willing R, Heß L, Mitschke J, Klemm N, Treiber J, Müller CA, Knecht S, Schwartz I, Weigt M, Bock M, von Elverfeldt D, Zaitsev M, Chekmenev EY, Hövener J-B, Martins AF, Schilling F, Reinheckel T, Schmidt AB (2025). *In vivo* molecular imaging of breast cancer metabolic heterogeneity using [1-
${}^{13}C$
]pyruvate-d_3_ hyperpolarized by reversible exchange with parahydrogen. Theranostics.

[bib1.bibx67] Picazo-Frutos R, Sheberstov KF, Blanchard JW, Van Dyke E, Reh M, Sjoelander T, Pines A, Budker D, Barskiy DA (2024). Zero-Field 
J
-Spectroscopy of Quadrupolar Nuclei. Nat Commun.

[bib1.bibx68] Pileio G (2010). Relaxation Theory of Nuclear Singlet States in Two Spin-
1/2
 Systems. Prog Nucl Mag Res Sp.

[bib1.bibx69] Pravdivtsev AN, Hövener J-B (2019). Simulating Non-linear Chemical and Physical (CAP) Dynamics of Signal Amplification By Reversible Exchange (SABRE). Chem Eur J.

[bib1.bibx70] Pravdivtsev AN, Ivanov KL, Kaptein R, Yurkovskaya AV (2013). Theoretical Study of Dipolar Relaxation of Coupled Nuclear Spins at Variable Magnetic Field. Appl Magn Reson.

[bib1.bibx71] Pravdivtsev AN, Yurkovskaya AV, Vieth H-M, Ivanov KL, Kaptein R (2013). Level Anti-Crossings are a Key Factor for Understanding para-Hydrogen-Induced Hyperpolarization in SABRE Experiments. ChemPhysChem.

[bib1.bibx72] Pravdivtsev AN, Yurkovskaya AV, Lukzen NN, Ivanov KL, Vieth HM (2014). Highly Efficient Polarization of Spin-
1/2
 Insensitive NMR Nuclei by Adiabatic Passage through Level Anticrossings. J Phys Chem Lett.

[bib1.bibx73] Pravdivtsev AN, Yurkovskaya AV, Lukzen NN, Vieth HM, Ivanov KL (2014). Exploiting Level Anti-Crossings (LACs) in the Rotating Frame for Transferring Spin Hyperpolarization. Phys Chem Chem Phys.

[bib1.bibx74] Pravdivtsev AN, Yurkovskaya AV, Vieth HM, Ivanov KL (2014). Spin Mixing at Level Anti-Crossings in the Rotating Frame Makes High-Field SABRE Feasible. Phys Chem Chem Phys.

[bib1.bibx75] Pravdivtsev AN, Ivanov KL, Yurkovskaya AV, Petrov PA, Limbach H-H, Kaptein R, Vieth H-M (2015). Spin Polarization Transfer Mechanisms of SABRE: A Magnetic Field Dependent Study. J Magn Reson.

[bib1.bibx76] Pravdivtsev AN, Buntkowsky G, Duckett SB, Koptyug IV, Hovener JB (2021). Parahydrogen-Induced Polarization of Amino Acids. Angew Chem Int Edit.

[bib1.bibx77] Put P, Pustelny S, Budker D, Druga E, Sjolander TF, Pines A, Barskiy DA (2021). Zero- to Ultralow-field NMR Spectroscopy of Small Biomolecules. Anal Chem.

[bib1.bibx78] Put P, Alcicek S, Bondar O, Bodek L, Duckett S, Pustelny S (2023). Detection of Pyridine Derivatives by SABRE Hyperpolarization at Zero Field. Commun Chem.

[bib1.bibx79] Pyper N (1971). Theory of Symmetry in Nuclear Magnetic Relaxation. Mol Phys.

[bib1.bibx80] Rayner PJ, Duckett SB (2018). Signal Amplification by Reversible Exchange (SABRE): From Discovery to Diagnosis. Angew Chem Int Edit.

[bib1.bibx81] Rodriguez GG, von Petersdorff-Campen C, Korchak S, Sucre O, Santi MD, Elsasser J, Mei R, Fries LM, Felger J, Markus A, Alves F, Glöggler S (2025). Biological 
J
-Coupling Spectroscopy at Low Magnetic Field. Small.

[bib1.bibx82] Schmidt AB, Eills J, Dagys L, Gierse M, Keim M, Lucas S, Bock M, Schwartz I, Zaitsev M, Chekmenev EY, Knecht S (2023). Over 20 % Carbon-13 Polarization of Perdeuterated Pyruvate Using Reversible Exchange with Parahydrogen and Spin-Lock Induced Crossing at 50 μT. J Phys Chem Lett.

[bib1.bibx83] Schwartz LJ, Stillman AE, Freed JH (1982). Analysis of Electron Spin Echoes by Spectral Representation of the Stochastic Liouville Qquation. J Chem Phys.

[bib1.bibx84] Shchepin RV, Birchall JR, Chukanov NV, Kovtunov KV, Koptyug IV, Theis T, Warren WS, Gelovani JG, Goodson BM, Shokouhi S, Rosen MS, Yen YF, Pham W, Chekmenev EY (2019). Hyperpolarizing Concentrated Metronidazole (
${\mathrm{NO}}_{2}$
)-
${}^{15}N$
 Group over Six Chemical Bonds with More than 15 % Polarization and a 20 Minute Lifetime. Chem Eur J.

[bib1.bibx85] Sheberstov KF, Chuchkova L, Hu Y, Zhukov IV, Kiryutin AS, Eshtukov AV, Cheshkov DA, Barskiy DA, Blanchard JW, Budker D, Ivanov KL, Yurkovskaya AV (2021). Photochemically Induced Dynamic Nuclear Polarization of Heteronuclear Singlet Order. J Phys Chem Lett.

[bib1.bibx86] Sjolander TF, Blanchard JW, Budker D, Pines A (2020). Two-Dimensional Single- and Multiple-Quantum Correlation Spectroscopy in Zero-Field Nuclear Magnetic Resonance. J Magn Reson.

[bib1.bibx87] Snadin AV, Chuklina NO, Kiryutin AS, Lukzen NN, Yurkovskaya AV (2024). Magnetic Field Dependence of the Para-Ortho Conversion Rate of Molecular Hydrogen in SABRE Experiments. J Magn Reson.

[bib1.bibx88] Stern Q, Sheberstov K (2023). Simulation of NMR spectra at zero and ultralow fields from A to Z – a tribute to Prof. Konstantin L'vovich Ivanov. Magn Reson.

[bib1.bibx89] Svyatova A, Skovpin IV, Chukanov NV, Kovtunov KV, Chekmenev EY, Pravdivtsev AN, Hovener JB, Koptyug IV (2019). ${}^{15}N$
 MRI of SLIC-SABRE Hyperpolarized 
${}^{15}N$
-Labelled Pyridine and Nicotinamide. Chem Eur J.

[bib1.bibx90] Teleanu F, Fabricant AM, Zhang C, Centers GP, Budker D, Barskiy DA, Jerschow A (2025). Nuclear Spin Relaxation in Zero-to Ultralow-field Magnetic Resonance Spectroscopy. arXiv [preprint].

[bib1.bibx91] Theis T, Ganssle P, Kervern G, Knappe S, Kitching J, Ledbetter MP, Budker D, Pines A (2011). Parahydrogen-Enhanced Zero-Field Nuclear Magnetic Resonance. Nat Phys.

[bib1.bibx92] Theis T, Ledbetter MP, Kervern G, Blanchard JW, Ganssle PJ, Butler MC, Shin HD, Budker D, Pines A (2012). Zero-Field NMR Enhanced by Parahydrogen in Reversible Exchange. J Am Chem Soc.

[bib1.bibx93] TomHon P, Gyesi J, Abdurraheem A, McBride S, Samoilenko A, Oladun C, Curran E, Pike M, Welch SD, Scofield S, Goodson BM, Sadagurski M, Theis T, Chekmenev EY (2025). Biocompatible SABRE Hyperpolarization of [1-
${}^{13}C$
]Ketoleucine for Cellular Metabolic Flux Sensing. Chem Eur J.

[bib1.bibx94] Van Dyke ET, Eills J, Picazo-Frutos R, Sheberstov KF, Hu Y, Budker D, Barskiy DA (2022). Relayed Hyperpolarization for Zero-Field Nuclear Magnetic Resonance. Sci Adv.

[bib1.bibx95] Vaneeckhaute E, Tyburn JM, Kempf JG, Martens JA, Breynaert E (2023). Reversible Parahydrogen Induced Hyperpolarization of 
${}^{15}N$
 in Unmodified Amino Acids Unraveled at High Magnetic Field. Sci Adv.

[bib1.bibx96] Xu J, Kircher R, Tretiak O, Budker D, Barskiy DA (2026). Quantum Magnetic 
J
-Oscillators. Nat Commun.

[bib1.bibx97] Zachrdla M, Turhan E, Bučková M, Hänsel-Hertsch R, Trantírek L, Kurzbach D (2025). Hyperpolarized NMR Reveals Low-Populated Folding Intermediates in DNA. J Am Chem Soc.

[bib1.bibx98] Zaiss M, Bachert P (2013). Exchange-Dependent Relaxation in the Rotating Frame for Slow and Intermediate Exchange–Modeling Off-Resonant Spin-Lock and Chemical Exchange Saturation Transfer. NMR Biomed.

[bib1.bibx99] Zheng L, Peng Q, Sun H, Deng J, Jiang Y, Xiong Y, Chen L, Cui X, Lin H, Chen Z, Wang X, Gao J (2026). SABRE Hyperpolarized Multichannel 
${}^{19}F$
 NMR for Sensitive Detection of Multiple Disease Marker Enzymes on a Benchtop NMR. Angew Chem Int Edit.

[bib1.bibx100] Zhukov IV, Kiryutin AS, Ferrage F, Buntkowsky G, Yurkovskaya AV, Ivanov KL (2020). Total Correlation Spectroscopy Across All NMR-Active Nuclei by Mixing at Zero Field. J Phys Chem Lett.

[bib1.bibx101] Zhukov IV, Kiryutin AS, Yurkovskaya AV, Blanchard JW, Budker D, Ivanov KL (2021). Correlation of High-Field and Zero- to Ultralow-field NMR Properties Using 2D Spectroscopy. J Chem Phys.

